# Emerging probing perspective of two-dimensional materials physics: terahertz emission spectroscopy

**DOI:** 10.1038/s41377-024-01486-2

**Published:** 2024-06-29

**Authors:** Yifei Wu, Yuqi Wang, Di Bao, Xiaonan Deng, Simian Zhang, Lin Yu-chun, Shengxian Ke, Jianing Liu, Yingjie Liu, Zeli Wang, Pingren Ham, Andrew Hanna, Jiaming Pan, Xinyue Hu, Zhengcao Li, Ji Zhou, Chen Wang

**Affiliations:** 1 State Key Laboratory of New Ceramics and Fine Processing, Key Laboratory of Advanced Materials of Ministry of Education, School of Materials Science and Engineering, Tsinghua University, 100084 Beijing, China; 2 Beijing Advanced Innovation Center for Integrated Circuits, 100084 Beijing, China

**Keywords:** Terahertz optics, Photonic devices

## Abstract

Terahertz (THz) emission spectroscopy (TES) has emerged as a highly effective and versatile technique for investigating the photoelectric properties of diverse materials and nonlinear physical processes in the past few decades. Concurrently, research on two-dimensional (2D) materials has experienced substantial growth due to their atomically thin structures, exceptional mechanical and optoelectronic properties, and the potential for applications in flexible electronics, sensing, and nanoelectronics. Specifically, these materials offer advantages such as tunable bandgap, high carrier mobility, wideband optical absorption, and relatively short carrier lifetime. By applying TES to investigate the 2D materials, their interfaces and heterostructures, rich information about the interplay among photons, charges, phonons and spins can be unfolded, which provides fundamental understanding for future applications. Thus it is timely to review the nonlinear processes underlying THz emission in 2D materials including optical rectification, photon-drag, high-order harmonic generation and spin-to-charge conversion, showcasing the rich diversity of the TES employed to unravel the complex nature of these materials. Typical applications based on THz emissions, such as THz lasers, ultrafast imaging and biosensors, are also discussed. Step further, we analyzed the unique advantages of spintronic terahertz emitters and the future technological advancements in the development of new THz generation mechanisms leading to advanced THz sources characterized by wide bandwidth, high power and integration, suitable for industrial and commercial applications. The continuous advancement and integration of TES with the study of 2D materials and heterostructures promise to revolutionize research in different areas, including basic materials physics, novel optoelectronic devices, and chips for post-Moore’s era.

## Introduction

**Fig. 1:**
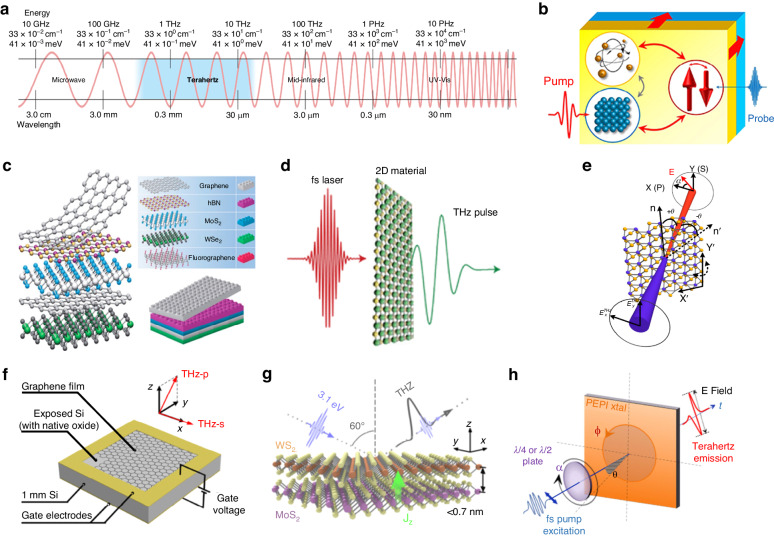
THz spectrum interacted with emerging 2D materials. **a** THz spectrum and its corresponding wavelength and energy. **b** The Interaction of THz on electrons, spins, and phonons within materials. **c** vdW heterostructure: based on typical 2D materials. **d** Schematic illustration of THz emission from 2D materials. **e** Schematic illustration of THz emission from layered MoS_2_ monolayer. **f** Active THz surface emission in Graphene/SiO_2_/Si heterostructure. **g** THz emission in WS_2_/MoS_2_ heterostructure by optically triggered interfacial current J_z_. **h** THz emission induced by Rashba splitting in 2D-phenethylammonium lead iodide (2D-PEPI) crystal. **a** Reprinted with permission from ref. ^1^. **b** Reprinted with permission from ref. ^15^. **c** Reprinted with permission from ref. ^40^. **d** Reprinted with permission from ref. ^52^. **e** Reprinted with permission from ref. ^53^. **f** Reprinted with permission from ref. ^54^. **g** Reprinted with permission from ref. ^56^. Fig **h**, reprinted with permission from ref. ^57^

Within electromagnetic spectrum, the THz region, historically spanning frequencies from 0.1 THz to 10 THz, has been referred to as the “terahertz gap” between microwaves and far-infrared radiation, posing challenges in generating and detecting radiation within this range (Fig. 1a)^1^. However, recent advancements in technologies such as optical rectification using femtosecond laser pulses, quantum cascade lasers (QCLs)^2^, tunable THz waveguide^3–5^ and free electron lasers^6^ have significantly expanded access to the THz part of the electromagnetic spectrum, overcoming previous limitations and creating new opportunities for research and applications in fields including THz spectroscopy, material science, communication, agriculture, environment, medical imaging, security screening and food^7–14^. Particularly in the field of material science, THz spectroscopy has been successfully employed to characterize different physical properties of materials, such as complex dielectric constant, refractive index, conductivities and so on. The unique frequency range of THz spectroscopy makes it well-suited for investigating intraband electrical transport and low-energy excitations in emerging materials. Its contact-free nature as a free-space optical technique eliminates the need for electrical contacts and minimizes material contamination or modification. Additionally, the ultrashort duration of sub-picosecond THz pulses enables time-resolved studies of ultrafast processes using techniques like TES, optical pump–THz probe (OPTP) spectroscopy or THz pump–THz probe (TPTP) spectroscopy, among which TES has emerged as a powerful tool for investigating materials’ electronic and optical properties. By harnessing the unique properties of emitted THz waves, researchers can delve into the intricate interplay between photons, charges, phonons, and spins (Fig. 1b)^15^, enabling the exploration of complex physical phenomena and intrinsic properties in materials. These advancements in THz technology not only bridge the historical ‘terahertz gap’ but also open new avenues for scientific research and practical applications, laying the groundwork for the next sections to discuss the specific uses and implications of these technologies in various fields.

Meanwhile, 2D materials have garnered significant attention for their extraordinary electronic, optical, and mechanical properties^16–30^. Graphene, transition metal dichalcogenides (TMDs), black phosphorus, quantum wells, and 2D electron gas, along with additional examples such as hexagonal boron nitride (hBN) and layered perovskite, stand out as prominent examples of 2D materials^31–39^. Due to their atomically thin structure, 2D materials exhibit high surface-to-volume ratios, enabling enhanced surface interactions and sensitivity to external stimuli, which open up new possibilities for sensing, catalysis, and energy applications. Furthermore, the integration and stacking of 2D materials in van der Waals (vdW) heterostructures have significantly enhanced artificial lattice structures, leading to the formation of novel material systems that exhibit unique and unprecedented physical properties (Fig. 1c)^40^. By stacking different 2D materials and controlling their stacking angles, one can engineer and tailor the electronic and optical properties that allow the discovery of intriguing phenomena such as superconductivity, interfacial charge transfer, interlayer exciton formation and so on. From an industrial perspective, notable progress has been achieved in the fabrication^41–44^, device development^45–47^, and industrial applications of 2D materials^48–50^. These advancements position 2D materials to have a pivotal role in the post-Moore’s law era of the chip industry^51^. Consequently, the development and integration of these materials into devices are essential for technological advancement and shaping the future of the semiconductor industry, surpassing the constraints of conventional silicon-based chips. To fully understand 2D material physics and use the full capabilities of those materials requires sophisticated characterization techniques, while the TES has been shown to be an excellent optical approach to studying 2D materials (Fig. 1d)^52^ with non-intrusive probing of systems. In principle, TES measures the emitted THz time domain electric field that contains both amplitude and phase information, which can be used to fully detect the structure and electrical properties of materials. For example, pioneering research has been conducted using TES to study 2D materials such as MoS_2_ monolayer (Fig. 1e)^53^, which reveals the unique play of surface depletion field-induced photocurrent surge in addition to optical rectification. In 2D heterostructures, interfacial effects dominate the nonlinear processes, such as active THz surface emission in Graphene/SiO_2_/Si heterostructures (Fig. 1f)^54^, THz wave modulation via metasurfaces in MoS_2_/Si heterostructures^55^, and interfacial current triggered THz emission in WS_2_/MoS_2_ heterostructures (Fig. 1g)^56^. Besides, the complex physical phenomena and mechanisms such as Rashba splitting (Fig. 1h)^57^ are also been deeply studied. Those different materials and heterostructures serve as fascinating platforms to hold different THz emission mechanisms, thereby enabling the exploration of complex physical phenomena and intrinsic material properties. Firstly, 2D materials provide high mobility and ultrafast carrier dynamics, enabling efficient generation and detection of THz waves. Secondly, the strong light–matter interaction in 2D materials allows for enhanced THz emission, making the spectroscopy more sensitive and accurate. Additionally, the flexibility and tunable electronic properties of 2D materials allow for the development of compact and versatile THz devices. This can lead to advancements in a wide range of applications, from security scanning and medical imaging to wireless communication and material characterization. The integration of 2D materials into THz spectroscopy thus opens up new avenues for exploring and exploiting the THz spectrum in both fundamental research and practical applications.

Hereby we comprehensively review TES and its application in studying 2D materials and heterostructures. Particularly we focus on the diverse nonlinear processes underlying THz emission in 2D materials and interfaces, such as optical rectification, photon-drag, high-order harmonic THz generation, and spin-to-charge conversion. By utilizing the capabilities of TES, researchers gain deeper insights into the electronic and optical properties of 2D materials, opening the way for progress in fields ranging from materials science to photonics, optoelectronics, and quantum technologies.

## Typical setups for THz spectroscopy

**Fig. 2:**
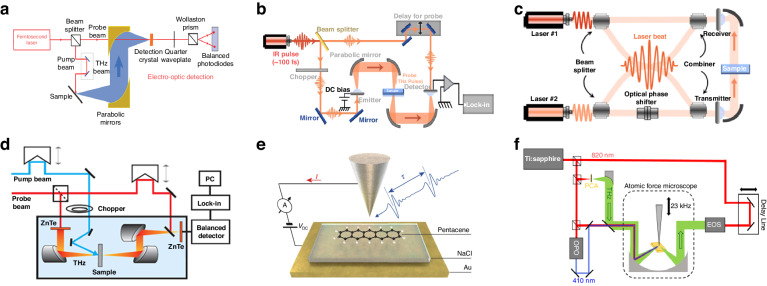
Schematic of typical experimental setups for THz spectroscopy. **a** Schematic illustration of typical TES set up. **b** Schematic illustration of typical THz-TDS set up. **c** Schematic illustration of typical THz-FDS set up. **d** Schematic illustration of typical TRTS set up. **e** THz spectroscopy combines with STM. **f** Schematic illustration of typical THz-SNOM set up. **a** Reprinted with permission from ref. ^58^. **b**, **c** Reprinted with permission from ref. ^65^. **d** Reprinted with permission from ref. ^68^. **e** Reprinted with permission from ref. ^72^. **f** Reprinted with permission from ref. ^78^

To investigate the structure and optoelectronic properties of 2D materials using THz spectroscopy, various experimental setups have been established. The most common setup for THz spectroscopy includes a femtosecond laser for generating THz wave and a detection part based on electro-optic (EO) sampling. As we know the TES utilizes macroscopic photocurrents to generate detectable THz pulses that provide information about the dynamics of current flow. This technique has emerged as a crucial tool for investigating the behavior of photocarriers under femtosecond laser excitation in sophisticated material systems. Figure 2a shows a specific setup for TES^58^. A Ti: Sapphire oscillator generates ultrashort pulses that induce THz emission when incident on the sample at a 45° angle. Gold-coated parabolic mirrors collect, collimate, and focus the THz radiation onto a ZnTe (110) EO crystal. The electric field of the THz wave induces a change in the refractive index of the crystal, modulating the polarization of a gating beam passing through the crystal, and the gating beam is then measured using a differential detector consisting of two photodiodes. The THz electric field waveform is measured as a function of time by varying the time delay between the pump and gating pulses. It’s important to note that a TES setup is a fundamentally broad concept, differing significantly from the specific setup mentioned previously. Depending on the systems to be studied, it can use various lasers with tuned wavelengths, and the measurement can be in either transmission or reflection geometry, and laser incident angle can be any angle, depending on the lattice structure and symmetry. The THz detector can use different nonlinear crystals depending on the THz intensity and bandwidth to be measured. TES has been employed in the investigation of semiconductors and extended to various other materials^59–61^. For instance, it was used to study aligned semiconducting carbon nanotubes, where the amplitude of THz emission correlated with the deposited pump energy and revealed the ultrafast dissociation dynamics of excitons^62^. The studies demonstrate the immense potential of TES in studying advanced material systems in the future. Moreover, TES serves as a valuable complement to THz time-domain spectroscopy (THz-TDS), through the spatial scanning of the pump-laser spot, it is possible to obtain localized information about the dynamics of photocarriers, achieving a spatial resolution limited only by the diameter of the pump-laser beam. The detailed exploration of TES, from its experimental setup to its diverse applications in studying material properties, underscores its significance in advancing our understanding of 2D materials and optoelectronics, paving the way for further innovations in material science.

In addition, THz-TDS is a widely used technique^63,64^, which utilizes ultrafast THz pulses for studying the interaction between THz radiation and matter. It involves measuring the temporal profile of THz pulses transmitted or reflected by materials to characterize their properties like complex refractive index. This non-destructive and contactless technique finds applications in diverse fields, including materials science, physics, biology, and telecommunications. A typical THz-TDS setup using photoconductive antennas or nonlinear crystals for both THz pulse generation and detection is shown in Fig. 2b^65^. A femtosecond infrared pulse is split into two, with one part generating the THz pulse and the other part used for detection via the EO sampling. This setup enables the characterization of materials in the THz range by measuring the transmitted THz waves for sample and reference. Specifically, photoconductive antennas (PCAs) are also widely used for detection, the process differs from EO sampling, though both methods aim to measure the THz electric field in the time domain. The THz-TDS allows phase-sensitive generation and detection of THz pulses, which directly provides the complex components of the material without the need for applying the Kramers–Kronig relations^66^, for example, magnetic susceptibility, optical conductivity, permittivity, etc., facilitating comprehensive investigations of different materials and phenomena. The THz frequency-domain spectroscopy (THz-FDS) system, utilizing continuous wave (CW) THz technology, offers precise frequency control and a higher dynamic range compared to the traditional THz-TDS, especially valuable for measurements requiring frequency sweeping and higher spectral resolution for sharp excitation probing. Figure 2c illustrates a typical THz-FDS setup, employing four-wave mixing, which achieves significantly better spectral resolution and dynamic range than the THz-TDS illustrated in Fig. 2b. In other aspects, by incorporating an optical pump path, the THz-TDS can be transformed into a so-called time-resolved THz spectroscopy (TRTS) technique, which enables the study of ultrafast carrier dynamics^67^. A specific TRTS system, depicted in Fig. 2d^68^, the pump beam is modified using a crystal to achieve the required wavelength and controlled fluence. THz probe pulses are created via optical rectification in a ZnTe crystal, and the THz radiation that passes through is detected using the EO sampling method in another ZnTe crystal. This setup allows for the investigation of the dynamics of photocarriers with high precision. By using TRTS, one can measure the changes in optical properties with sub-picosecond resolution, which provides insights into the relaxation dynamics of electronic states in the sample, facilitating the study of ultrafast quantum dynamics in materials^67,69,70^.

Based on TES, by integrating THz spectroscopy with other mature complementary characterization techniques, more comprehensive range of material characterization becomes achievable, enabling the exploration of local properties in a wider range of materials such as semiconducting nanostructures and 2D materials. For instance, the combination of THz spectroscopy with scanning tunneling microscopy (STM) enables the attainment of high spatiotemporal resolution. The integration of THz-STM facilitates the study of ultrafast electron dynamics at sub-nanoscale spatial dimensions and sub-picosecond temporal resolutions^71–73^. This emerging technique has garnered significant attention due to its potential for uncovering intricate details and phenomena in low-dimensional materials. Figure 2e depicts the experimental setup, which directs two THz pulses with an adjustable delay time into a STM junction^72^. This junction comprises a tungsten tip and a pentacene molecule, isolated from the Au (110) substrate by a NaCl film layer. The resulting current from the THz pulses was measured as a change in the direct current tunnel current. The bias voltage represents the voltage difference between the metal substrate and the tip. Positive currents signify electron tunneling from the tip into the sample. Yoshida et al. employed THz-STM to study photoinjected free electrons in a C_60_ multilayer film on an Au (111) substrate^74^. This work successfully visualized electron motion triggered by variations in the lowest unoccupied molecular orbital. This method will be crucial for evaluating local electronic structures and dynamics in 2D materials and semiconducting nanodevices. As the only technique which is capable of taking single-electron ultrafast movies at atomic resolution, THz-STM still has a lot of room for improvement, such as a higher signal-to-noise ratio^75^. Furthermore, the introduction of scattering-type scanning near-field optical microscopy (s-SNOM) has revolutionized subwavelength optics^76,77^. By coupling electromagnetic radiation to a subwavelength metal tip near a surface, s-SNOM allows the measurement of scattered radiation in the far field. Figure 2f shows the first s-SNOM measurement using a blue light (photon energy exceeds 3 eV) incident wave in the form of blue-pulse-induced THz emission^78^, high-energy pump photons enable strong THz emission from bulk crystalline silicon via two-photon excitation above the wide direct bandgap. Nanoscale spatial resolution is achieved in laser THz emission microscopy (LTEM) using femtosecond pulses at 410 nm and inducing THz emission with a sharp metallic atomic force microscope (AFM) tip. The setup involves generating near-infrared, blue light, and THz beams separately and detecting scattered or emitted THz pulses using EO sampling. Pizzuto et al. obtained the first near-field LTEM image of Si and compared it to normal THz s-SNOM results, which opens possibilities for applying s-SNOM to wide-bandgap materials and expanding LTEM spectroscopy in directly observing charge carrier properties. The integration of THz emission spectroscopy with advanced techniques like THz-STM and s-SNOM represents a significant leap in material science, offering unprecedented insights into the ultrafast and nanoscale dynamics of materials, and sets the stage for future developments in the field of high-resolution material characterization.

In near-field measurements, the pump and probe spots are typically localized to the scattering probe’s tip. However, to study non-local phenomena and obtain direct information about non-local effects, the use of two optical beams has been explored^75^. Pizzuto et al. conducted a non-local near-field pump-probe experiment on undoped bulk GaAs and GaAs nanowires. By photoexciting the sample at a laterally shifted location from the metallic probe tip using an ultrafast near-infrared pulse and probing with a broadband (0.2–2 THz) pulse scattered from the metal tip, researchers observed time-shifted conductivity responses consistent with carrier mobility simulations. This experiment confirmed the anisotropy of undoped GaAs nanowires and demonstrated the potential of the technique for studying nanoscale anisotropy, which is not feasible in the far field. The exploration of non-local phenomena through advanced THz spectroscopy techniques, as exemplified in the studies of bulk GaAs and GaAs nanowires, underscores the versatility and depth of THz analysis. The various THz spectroscopy and extended techniques mentioned facilitate comprehensive investigations into the structure and properties of materials. These advancements in THz spectroscopy open up new possibilities for studying ultrafast quantum effects, characterizing nanoscale structures, and exploring nonlocal effects, ultimately advancing our understanding of material physics and facilitating the development of advanced technologies. The coming sections will focus on the discussion of THz emission spectroscopy and review its application in studying 2D materials and their nanostructures.

## THz emission from non-centrosymmetric 2D materials

**Fig. 3:**
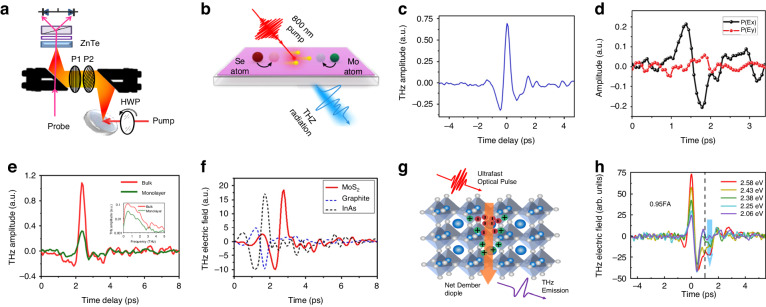
THz emission from non-centrosymmetric 2D monolayers. **a** Schematic illustration of experimental set up for THz emission from layered 2D monolayer. **b** Schematic illustration of the resonant optical rectification mechanism for MoSe_2_ THz emission. **c** Time-domain representation of typical THz emissions generated from WSe_2_. **d** THz emission electric field amplitudes from monolayer WS_2_ excited by p-polarization pump beam, with black and red lines representing *E*_x_ and *E*_y_ components, respectively. **e** Time-domain representation of typical THz emission (inset: frequency domain) generated from layered bulk (red solid line) and monolayer (green solid line) MoSe_2_. **f** Time-domain representation of generated THz emission pulses from layered MoS_2_ (red solid curve), graphite (blue dash curve), and InAs crystal (black dash curve). The THz emissions from MoS_2_ and graphite are amplified 15 times for better visibility. **g** Schematic illustration of the THz emission mechanism in FAPbI_3_, showcasing the polaron emission from the photo-Dember effect. **h** THz time-domain emission spectra of 0.95FA under different pump photon energies (carrier concentration *n* = 1.25 × 10^18 ^cm^−3^). **a**, **f** Reprinted with permission from ref. ^80^. **b**, **e** Adapted with permission from ref. ^84^. Copyright 2024 American Chemical Society. Fig **c**, reprinted with permission from ref. ^82^. **d** Reprinted with permission from ref. ^83^. **g**, **h** Reprinted with permission from ref. ^85^

Building on the foundation of THz emission spectroscopy’s versatility as discussed earlier, we now delve into its application in the realm of 2D materials, particularly in the study of layered TMDs known for their exceptional optical properties. Layered TMDs, such as WS_2_, WSe_2_, MoS_2_ and MoSe_2_, are the type of 2D materials widely known for their remarkable optical properties. Unlike gapless graphene, TMDs exhibit non-centrosymmetric lattice structure and possess tunable band gaps that can transition from indirect to direct band gaps at monolayer limit^31^. Figure 3a shows a typical experimental setup for THz emission from a layered 2D monolayer. The layered MoS_2_ crystal generates efficient linearly polarized THz radiation (0.1–3.5 THz) under femtosecond laser excitation and the THz amplitude exhibits a linear dependence on pump fluence, indicating a second-order nonlinear process^79,80^. In principle, under femtosecond laser, charges in the materials are accelerated by the surface field in the surface-charge region. This acceleration leads to the generation of THz emission, which contains valuable information such as amplitude, polarization, and phase. Furthermore, THz surface emission spectroscopy enables non-contact observation of surface optical-physical processes with high temporal and frequency resolution. This technique has been successfully applied to investigate surface phenomena in various materials, especially in 2D materials. It is important to note that the THz emission mechanisms can differ between bulk materials and monolayer materials^81^. For example, the depletion field effect dominates in bulk WSe_2_^82^, while the surface optical rectification dominates in bulk MoS_2_^80^_,_ and the circular optical rectification effect dominates in monolayer WS_2_^83^. Unlike bulk materials, monolayer materials exhibit strong nonlinear optical effects due to inversion symmetry breaking.

Commonly, optical rectification is one of the primary mechanisms in generating THz radiation using the second-order nonlinear effect. Taking MoSe_2_ as an example (Fig. 3b), under an 800 nm pump excitation, the movement of positive and negative charge centers results in a change in the charge density, which leads to an alteration in polarization density^84^. Because the displacement of Mo and Se atoms occurs at the bond length level, and this process unfolds on a femtosecond timescale, the frequency of the excited electromagnetic wave falls within the THz range. Similarly, excitation of THz electromagnetic waves has been observed in other 2D materials, such as WSe_2_ (Fig. 3c)^82^, indicating that optical rectification is mostly the dominant mechanism of THz emission in non-centrosymmetric monolayer materials. External conditions such as the polarization excitation in different directions of the pump can also impact THz excitation. For example, in WS_2_, when pump beams are in the s-polarization direction and p-polarization directions, they have an impact on the amplitude and phase of the excited THz radiation components, *E*_*x*_ and *E*_*y*_. The amplitude of *E*_*x*_ is greater than that of *E*_*y*_, indicating that the THz energy is mainly concentrated in the *E*_*x*_ component, and the phase of *E*_*x*_ and *E*_*y*_ is roughly opposite (Fig. 3d)^83^. Therefore, circularly polarized excitation generates elliptically polarized THz waves (max. ellipticity ≈ 0.52) through circular optical rectification. In addition, polarized THz radiation can be easily controlled with the quarter-wave plate (QWP), which makes THz emission more controllable and advance the understanding of THz radiation properties and polarization control in 2D materials. The physical reasons for the different THz emission phenomena between monolayer and bulk materials are worth studying. Fan et al. have further compared the THz radiation from bulk MoSe_2_ and monolayer MoSe_2_. Figure 3e shows the corresponding time-domain and frequency-domain spectra^84^. In the time-domain spectra, the THz amplitude of bulk materials is nearly four times that of monolayer materials due to the larger surface depletion field in bulk materials. In the frequency-domain spectra, the frequency range of radiation emitted by bulk materials is approximately 0–5 THz, while that of monolayer materials is 0–4 THz (Fig. 3e inset). For the bulk MoSe_2_, it has the point group structure of P_63_/mmc Space group and D_6h_. This central symmetry will theoretically make it lose the second-order nonlinear effect. However, it is noted that the surface of the bulk material only has the periodicity in the *XY* direction, and the periodicity in the Z direction is broken, leading to the point group structure degenerating to D_3h_, affecting the nonlinear coefficient leading to the generation of THz radiation. Although the surface of monolayer material and bulk material is similar, the width of the space charge region on the surface of bulk materials is much larger than that of monolayer materials. The presence of the space charge region induces the generation of transient photocurrent, and the first-order derivative of photocurrent on time determines the amplitude of THz radiation. Moreover, in bulk MoSe_2_, the influence of surface field-induced photocurrent on THz amplitude is greater than that of surface optical rectification on THz amplitude. Therefore, the amplitude of THz emission from bulk MoSe_2_ is approximately four times greater than that of monolayer MoSe_2_. Additionally, 2D monolayer nanomaterials exhibit notable surface and interface effects, in-plane dipole effects, and strong spin-orbit coupling, leading to complex and intriguing nonlinear optical processes in THz emission compared to bulk materials. This analysis of THz emission mechanisms in both monolayer and bulk materials, particularly in the context of MoSe_2_ and WS_2_, not only highlights the intricate interplay of physical processes governing THz radiation but also underscores the unique properties of 2D monolayer materials, setting a foundation for further exploration into their complex nonlinear optical behaviors and potential applications.

To explore the THz emission mechanism of different 2D materials, researchers have compared the THz emission graphite, MoS_2_, and InAs. In graphite, the presence of inversion symmetry in its layered structure prevents the occurrence of optical rectification. New mechanisms, such as the photon-drag mechanism, have been found to be responsible for its THz emission. For InAs, the carriers’ effective mass and mobility are significantly different. The effective mass of holes is more than ten times that of electrons, and the hole mobility is less than 1% of the electron mobility, while the effective mass and mobility of electrons and holes in MoS_2_ are in the same order of magnitude, which is common in 2D TMDs. This difference in InAs allows it to easily emit strong THz radiation by the photo–Dember effect, where the different mobility of electrons and holes leads to the destruction of surface symmetry, generating a Dember electric field. Under the modulation of the diffusion electric field and the Dember electric field, the excited electrons and holes form rapidly changing dipoles perpendicular to the surface and emit THz radiation. In addition, because the penetration depth of infrared light in MoS_2_ is greater than that of the other two materials, there will be a period shift in the time-domain spectrum (Fig. 3f)^80^. In addition to the 2D materials mentioned above, researchers have also observed THz emission in quasi-2D hybrid organic-inorganic perovskite (HOIPs) thin films via the photo-Dember effect (see Fig. 3g)^85^. The larger the laser photon energy, the larger the amplitude of the corresponding main peak of the THz wave. As shown in Fig. 3h, when the laser photon energy is greater than 2.43 eV and the time is 0.5 ps, the trend of these curves changes, the researchers illustrated two different polaron modes in the THz emission spectrum are existed, one dominated by inorganic sublattice vibration (P1) and the other involving the A-site cation (P2). This THz emission mode, which changes with the increase of excited photon energy, deserves further study. This exploration of THz emission mechanisms across a spectrum of 2D materials, including graphene, MoS_2_, InAs, and hybrid organic-inorganic perovskites, not only reveals the diverse underlying physical processes like the photo-Dember effect and photon-drag mechanism but also highlights the potential for further investigation into the complex interplay of material properties and THz emission characteristics, paving the way for new discoveries and applications in the field of nanotechnology.

Overall, the study of THz emission mechanisms in various monolayer (quasi-2D) materials provides insights into the complex processes involved in THz emission generation and helps us understand the unique properties of different materials in the THz regime. The explanation of these mechanisms allows researchers to unravel the 2D material physics underlying THz emission. In addition to investigating the THz emission mechanisms, there is ongoing research focused specifically on TMD-based THz emitters. These studies primarily aim to optimize and produce various types of controllable TMDs that can be utilized in THz applications. By engineering TMDs for specific THz applications, researchers can harness the unique properties of these materials and tailor them to meet the requirements of different technological advancements. We believe this ongoing research in TMD-based THz emitters holds great promise for the development of advanced THz devices and systems in the future.

## THz emission originated from the photon-drag effect

**Fig. 4:**
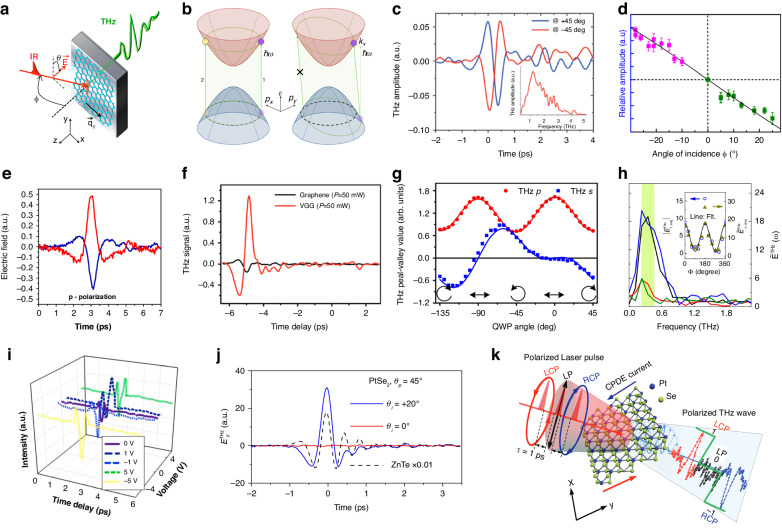
THz emission from photon-drag effect. **a** Schematic diagram of photon-drag induced THz emission from graphene, pumped by a femtosecond optical pulse. **b** Comparison between vertical transitions and nonvertical transitions (e.g., from photon-drag). Dashed black contours represent the Fermi surface and green line denotes the energy-momentum-conserving contours. **c** Spectra in time domain and frequency domain of the emitted THz electric field from monolayer graphene, with incidence angle of opposite signs. **d** Peak-to-peak amplitude of THz electric field emitted from graphene, as a function of the incidence angle. **e** THz emission spectra in time-domain obtained from a monolayer graphene sample with a p-polarized pump beam. Red lines, excited from graphene side; blue lines, excited from the substrate side. **f** Comparison of THz emission from VGG and monolayer graphene with a pumping power of 50 mW. **g** Dependence of the emitted THz peak-valley value on the QWP angle observed from VGG. **h** THz-wave magnitudes in frequency domains. Inset: average value of the shaded regions as a function of representative azimuth angles. **i** Electrically enhanced THz pulses from SnSe_2_ crystal. **j** THz emission signal from the PtSe_2_ thin film as a function of time delay under the linear laser pump, compared to the signal from the 0.5-mm-thick ZnTe crystal. **k** Schematic illustrations of the single-pulse ternary encoding of the polarized THz radiation. **a**, **d** Reprinted with permission from ref. ^86^. **b** Adapted with permission from ref. ^87^. **c** Reprinted with permission from ref. ^88^. **e** Reprinted with permission from ref. ^89^. **f** Reprinted with permission from ref. ^90^. **g** Reprinted with permission from ref. ^91^. **h** Reprinted with permission from ref. ^92^. **i** Reprinted with permission from ref. ^93^. **j** Reprinted with permission from ref. ^94^. **k** Reprinted with permission from ref. ^95^

THz emission, mainly from the second-order nonlinear process, is commonly expected to happen in non-centrosymmetric crystals. However, in centrosymmetric materials, THz radiation can also be generated by introducing additional asymmetry, such as inversion symmetry broken by light-induced quasiparticles, surface effect, or photothermal effect, etc. Among these approaches, the photon-drag effect has been studied as an important mechanism which utilizes the incoming beam with a tilted incident angle. Such an effect has been used for the generation of THz emission in graphene. As sketched in Fig. 4a, the femtosecond optical pump pulse illuminating at oblique incidence induces nonzero shift current dipole arising from an asymmetric distribution of nonthermal electron and hole population even in centrosymmetric multilayer graphene, giving rise to THz emission covering 0.1–4 THz^86^. This nonzero shift current dipole arises from a nonvertical transition between the valence and conduction bands due to the transferring of the finite in-plane photon momentum to the electron-hole pairs (Fig. 4b)^87^. It is worth noting that the polarity of the signals from graphene reverses for opposite incident angle (Fig. 4c)^88^, corresponding to an opposite shift current dipole caused by the opposite in-plane photon momentum. This relationship is further confirmed by varying the incident angle and comparing the peak-to-peak amplitude of the emitted THz electric field normalized by (1 − r) (r is the amplitude Fresnel coefficient of graphene) (Fig. 4d)^86^. Such mechanism based on photon-drag is similarly validated in another work with the surface photogalvanic effect excluded, in which the polarization of the laser beam can modulate the THz emission intensity^89^. Concretely, the excitation using a p-polarized beam from the substrate side reverses the polarity of the generated THz wave (Fig. 4e).

For improved efficiency and extra tunability of the photon-drag effect, THz emission from vertically grown graphene (VGG) is achieved. Figure 4f compares the emitted THz signal from VGG with monolayer graphene, the enhanced signal is attributed to the light-matter interaction enhancement and the more intensive photoexcited carriers in the VGG structure^90^. Additionally, the enhanced efficiency also enables prominent detection of circular photon-drag effect (CPDE). Helicity-dependent THz emission is observed in VGG by tuning the QWP polarization angle^91^, which is confirmed as the result of the combined contribution of CPDE and linear photon-drag effect (LPDE) with different weights As exhibited in Fig. 4g, there is a regular cycle of change in the peak-valley values of THz p components in the form of cos 4*φ* (*φ is* QWP polarization angle) due to the dominance of CPDE, while the amplitudes of THz s-components exhibits irregular variation curves as the weight of CPDE and LPDE changes with *φ*.

Apart from VGG, THz emission with modulated amplitude and phase is also demonstrated in layered selenides like Bi_2_Se_3_ and SnSe_2_. As an illustration, the polarized light excitation in the Bi_2_Se_3_ film could induce photon-drag effect (Fig. 4h)^92^, in which an apparent periodicity of the azimuth angles is observed (shown in the inset), attributed to the intrinsic symmetry of the Bi_2_Se_3_ crystal. Further research on SnSe_2_ demonstrated that applying a constant electric field is an effective way to enhance the intensity of photon-drag induced THz radiation to 123% (Fig. 4i)^93^.

Recently, PtSe_2_ attracted attention as an emerging 2D platform with a centrosymmetric crystal structure, in which THz emission could be observed when the incident angle (θ_i_) of the pump pulses is not zero (Fig. 4j)^94^. The emission efficiency through photon-drag mechanism is reported up to 5.2 × 10^10^ (defined as E/Fd, where E is the maximum THz electric field, F is the pump fluence, and d is the penetration depth for 800-nm light or the thickness of the sample), as a result of its particle-hole asymmetric band structure. For more specific applications, such THz emission from PtSe_2_ with polarization dependence was demonstrated to realize ultrafast ternary encoding as a new detection model (Fig. 4k). The positive or negative polarities of THz pulse signals were generated by left-circularly polarized or right-circularly polarized laser excitations, which was coded as 1 or −1, respectively. In contrast, when using a linearly polarized laser to excite, no THz pulse signals were detected which was coded as zero^95^. Such THz coding function based on photon-drag effect spreads the non-contact modulation capability and the application prospect in THz emission applications.

## THz emission from heterostructures

2D materials have the advantage of easy integration with traditional optoelectronic materials, systems, and devices, leading to novel phenomena and applications, especially in the THz regime. Integration is achieved through direct growth or thin-film transfer methods. The heterostructures can be usually distinguished into two types: one for vdW heterostructures are composed of different 2D materials^96,97^ and the other is heterogeneous integration with traditional materials and devices^98^.

**Fig. 5:**
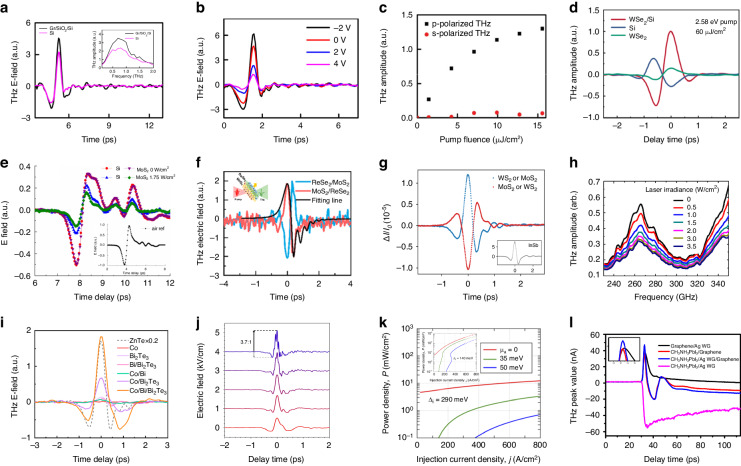
THz emission from heterostructures. **a** Gr/SiO_2_/Si and Gr/Si heterostructures’ p-polarized THz emission in time domain and frequency domain (inset) with no gate voltage. **b** Gr/SiO_2_/Si and Gr/Si heterostructures’ THz emission at gate voltage -2, 0, 2, and 4 V, respectively. **c** Graphene/Si Schottky junction p- and s- polarized THz emission amplitude with respect to pump fluence. **d** WSe_2_/Si heterostructure THz pulse waveform compared with that of Si and monolayer WSe_2_ under a femtosecond pulse (2.58 eV and 60 μJ/cm^2^). **e** MoS_2_/Si heterostructure THz wave in time-domain from Si metasurface and MoS_2_ metasurface in p-polarization state with and without photon pumping (inset is the air reference). **f** ReSe_2_/MoS_2_ and MoS_2_/ReSe_2_ heterostructures’ THz electric field waveforms (inset: schematic diagram of TES for ReSe_2_-MoS_2_ heterostructure) under 800 nm pump excitation (black line for the simulated curve using the photocurrent model). **g** WS_2_/MoS_2_ and MoS_2_/WS_2_ heterostructures THz electric field waveforms under 3.1 eV femtosecond pulses (Inset: calibration by a bulk InSb crystal under identical conditions). **h** THz spectrum analysis of CsPbBr_3_ perovskite quantum dots heterostructures under varying laser intensity irradiances. **i** Co/Bi(n)/Bi_2_Te_3_ heterostructures’ THz waveform compared with those of ZnTe*0.2, Co, Bi_2_Te_3_, Bi/Bi_2_Te_3_, Co/Bi, Co/Bi_2_Te_3_ films. **j** GaAsSb_0.13_/In_0.141_Ga_0.859_As heterostructure THz waveforms under pump-pulse durations from 140 to 30 fs from red to blue lines. the output THz power P generated by the GL/MoS_2_ (**k**) EDs (inset: GL/b-P) as a function of j. **l** Temporal evolution of pump-induced THz waveform peak alterations in various samples: graphene/Ag wire grid (black line), CH_3_NH_3_PbI_3_/graphene (red line), CH_3_NH_3_PbI_3_/Ag wire grid/graphene (blue line), and CH_3_NH_3_PbI_3_/Ag wire grid (pink line). The inset in the upper left shows an enlarged view of the peak region. **a**, **b** Reprinted with permission from ref. ^54^. **c** Reprinted with permission from ref. ^99^. **d** Reprinted with permission from ref. ^98^. **e** Reprinted with permission from ref. ^55^. **f** Adapted with permission from ref. ^100^, Copyright 2024 American Chemical Society. **g** Reprinted with permission from ref. ^56^. **h** Reprinted with permission from ref. ^101^. **i** Reprinted with permission from ref. ^103^. **j** Reprinted with permission from ref. ^104^. **k** Adapted with permission from ref. ^97^. **l** Reprinted with permission from ref. ^105^

As one of the typical 2D materials, Graphene has long been used to construct heterostructures to emit THz. It is commonly combined with SiO_2_ and Si bases for the on-chip industry. In a study by Yao et al., a noninvasive and efficient method was applied to investigate electric-field-induced optical rectification that indicates the correlation between built-in interface potential and THz generation. Without applying a gate voltage, Gr/SiO_2_/Si generates stronger THz than Si (Fig. 5a)^54^, which is mainly from the photocarriers with the picosecond response in the interface layer. When applying a negative gate voltage, THz generation is enhanced, where THz amplitude is three times higher when V_g_ = −2V compared with V_g_ = 2 V (Fig. 5b). This indicates that modifying the interface depletion layer can tune THz generation with gate voltage applied. Graphene/Si Schottky junction (GSSJ) is the efficient THz interfacial emission structure under the gate voltage.

Similar to monolayer materials, the polarization of heterojunction THz emission has also been studied. The orthogonal components of the induced THz field include p- and s-polarized THz emission, with the amplitude of s-polarized emission being much lower than that of p-polarized THz radiation (Fig. 5c)^99^. The investigation of THz emission in heterostructures related to TMDs has garnered significant attention. Compared with WSe_2_ only, WSe_2_/Si THz emission amplitude is ~10 times larger under a 1.65 eV laser pump (Fig. 5d)^98^. Additionally, THz modulators based on other TMDs-Si heterostructures, such as MoS_2_-Si, have also been fabricated and investigated. Since MoS_2_ sheets are nearly transparent for THz waves, THz emission amplitudes of MoS_2_ and Si metasurfaces are nearly the same when no laser is pumping. Compared to the reference signal in air, the pulse broadening and deformation are attributed to the resonances within the metasurface structure. Under laser pumping of 1.75 W/cm^2^, the amplitudes of THz emission from both metasurfaces decline, and the amplitude of THz emission from MoS_2_ decreases even more. This phenomenon can be attributed to the enhanced modulation depth of the Si/MoS_2_ configuration, leading to more photocarrier generation compared to a purely Si surface (Fig. 5e)^55^.

The heterostructures combined with TMDs and other 2D materials have also been widely studied in the field of THz emission. Krihna et al. applied hBN/WS_2_ heterostructure to investigate THz photoconductivity and photocarrier dynamics with interfacial dipole formation at the interface between insulator and semiconductor^96^. Other vdW heterostructures such as ReSe_2_/MoS_2_ heterostructure have also been investigated using THz emission (Fig. 5f inset). As shown in Fig. 5f, THz radiation for both ReSe_2_/MoS_2_ and MoS_2_/ReSe_2_ direction last for 300 fs^100^. In this study, various stacking orders and measurement techniques were employed to investigate photocarrier dynamics. Reversal of the emitted THz pulse was observed when the stacking order was interchanged, indicating that THz emissions arise from the plane current, and the frequency spectrum of the waveforms exhibited a peak at 1.0 THz with a bandwidth of 2.5 THz. A similar phenomenon has also been observed in WS_2_/MoS_2_ heterostructure that emits much stronger THz emission than pure WS_2_ or MoS_2_, indicating the interfacial charge transfer dominates the THz emission process^56^ (Fig. 5g).

Perovskite heterostructures, going beyond the realm of common heterostructures, have garnered significant interest in scientific research. These perovskite devices are particularly notable for their high modulation efficiency, rapid response times, cost-effectiveness, ease of integration, and stability. Specifically, the amplitude spectrum of the CsPbBr_3_ perovskite quantum dots combined with Si under various laser intensity irradiances is illustrated in Fig. 5h^101^. Additionally, the spectral transmission of the CsPbBr_3_ perovskite quantum dot heterostructure was meticulously measured using a Backward Wave Oscillator (BWO). Although it is not based on TES but rather TRTS method, there are still abundant physical phenomena worth exploring. These measurements were taken both with and without the application of an external 450 nm pump laser excitation, across a frequency range from 0.23 to 0.35 THz. To delve deeper into the modulation mechanism, researchers analyzed the optical constants of the CsPbBr_3_ perovskite quantum dots/Si. This analysis was conducted through time-frequency spectral measurements using the BWO under a range of laser irradiances. The findings revealed that the CsPbBr_3_ perovskite quantum dot heterostructure boasts a modulation speed of 2.5 MHz and a modulation depth of 45.5%. These impressive performance metrics strongly suggest that this heterostructure could be an excellent candidate for future applications in practical THz wave communication systems.

In addition to 2D materials, heterostructures composed of traditional materials also have great potential in THz emission. Kampfrath et al. demonstrated that laser pulses irradiating heterostructures composed of a ferromagnetic iron layer and a non-magnetic metal (ruthenium or gold) layer drive ultrafast spin currents and generate THz emission. This discovery indicates the great potential of spintronic THz emitters (STE) as strong THz sources^102^. Based on these findings, other heterostructures are also being utilized for THz emission. For example, Co/Bi(n)/Bi_2_Te_3_ multi-material stacking heterostructure is found to emit strong THz radiation. Adding (or reducing) a certain layer of material can cause a sharp change in the efficiency of heterojunction THz emission. Compared to the negligible THz signal from Bi_2_Te_3_, Bi/Bi_2_Te_3_, or Co/Bi films, strong THz emission is found from Co/Bi_2_Te_3_, Co/Bi/Bi_2_Te_3._due to the spin-to-charge conversion process (Fig. 5i)^103^. Significantly, the amplitude of the THz signal emitted from the Co/Bi/Bi_2_Te_3_ heterostructure can reach up to 198% of that emitted by devices lacking an intercalated Bi film.

Notably, shift current is another strategy to emit THz radiation in the heterostructures. In the GaAsSb_0.13_/In_0.141_Ga_0.859_As heterostructure, lower pump-pulse duration leads to an increase of the peak field of THz transients from 0.51 to 1.1 kV cm^−1^. On the other hand, the width of the field crest Δτ decreases with the pump-pulse duration from 70 fs to 20 fs (Fig. 5j)^104^. In addition to the above-mentioned combination of Graphene and traditional materials to build heterostructures, Graphene also builds heterostructures with other 2D materials. For example, in 2020, graphene-layer/black phosphorus (GL/BP) and GL/MoS_2_ heterostructures are theoretically expected to be THz emitting diodes (THz-EDs) with lateral hole and vertical electron injection. The increasing injection current density may lead to stronger output power in both heterostructures (Fig. 5k)^97^. The increase is correlated with doping level (μ_a_), and undoped GL is shown to be significantly boosted by injection current. It is noteworthy to clarify that, in this research, the term “injection current” pertains to an electrical means of THz emission, distinct from the optical counterpart. Although further experimental verification needs to be completed, this work has provided a feasible method for fabricating the more advanced THz emission devices based on 2D materials.

Researchers are increasingly focusing on complex heterostructures, particularly those incorporating a variety of materials. A notable example is as shown in Fig. 5l^105^, the CH_3_NH_3_PbI_3_/graphene heterostructure, where under an 800 nm, 288 mW pump beam, the THz peak signal initially rises then falls to a negative value, indicative of photo-enhanced transparency similar to monolayer graphene’s hot-carrier effect. This effect increases graphene’s chemical potential and temperature, while carrier−carrier scattering reduces electrical conductivity. In the CH_3_NH_3_PbI_3_/Ag wire grid (Ag WG)/graphene sample, the Ag wire grid boosts pump beam absorption, enhancing photogenerated carrier efficiency and transient THz transmittance. This sample’s THz signal recovers quicker than single-layer graphene’s, due to faster hot-carrier cooling and photoexcited carrier accumulation in the CH_3_NH_3_PbI_3_ layer. Furthermore, the sample exhibits a delayed negative THz signal, attributed to the long carrier migration distance in the CH_3_NH_3_PbI_3_ layer and the transfer of hot carriers from graphene to perovskite, thereby enhancing photoconductivity. The total THz transmission reflects the interplay between graphene and CH_3_NH_3_PbI_3_ materials. The Ag wire grid aids in electron transfer in CH_3_NH_3_PbI_3_, promoting effective electron–hole separation and efficient charge collection on graphene. This innovation opens new ways for THz wave modulation and visible light detection applications.

In summary, the investigations of THz emission from heterostructures have revealed their remarkable potential for generating and controlling THz radiation. Whether traditional material heterostructures or vdW heterostructures, these materials have demonstrated efficient THz emission through optical rectification, interfacial dipole formation, shift current, and spin-to-charge conversion which will be discussed in detail in the following section. The manipulation of gate voltages, modulation depths, and shift currents allows for tunability and enhancement of THz generation. Moreover, utilizing heterostructures opens up possibilities for practical applications and offers a versatile platform for exploring and harnessing the potential of THz radiation in various fields, paving the way for advancements in THz technology.

## High-order harmonic THz generation

**Fig. 6:**
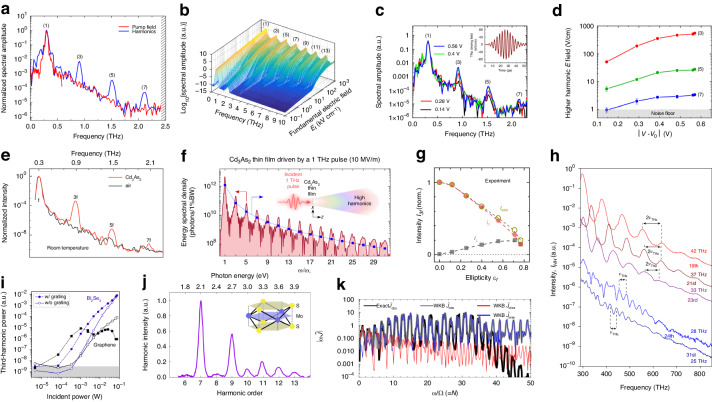
High-order Harmonic THz generation. **a** Spectrum of the pump field and THz harmonics emission from graphene. **b** Calculation of higher-order harmonic generation in graphene using the thermodynamic mechanism. **c** Spectra of the THz pump field and HHG modulated by the gate voltage. **d** maximum of the harmonic electric field as a function of the gate voltage. **e** Spectrum of THz HHG in Cd_3_As_2_. **f** Extreme harmonics up to 31st order generated in Cd_3_As_2_ at modest pump field strengths. **g** Intensity of the third-order THz harmonics from Cd_3_As_2_ as a function of driving pulse ellipticity. **h** Intensities of THz HHG form surface ($\nu \le$28 THz) and bulk ($\nu \ge$35 THz) of Bi_2_Te_3_. **i** THz harmonic power in Bi_2_Se_3_ and graphene as a function of incident power. **j** 6th to 13th harmonics spectrum from monolayer MoS_2_ with a fundamental field centered at a photon energy of 0.30 eV. **k** HHG spectrum for the oscillating field with analytical WKB theory. **a**, **b** Reprinted with permission from ref. ^106^. **c**, **d** Reprinted with permission from ref. ^107^. **e** Reprinted with permission from ref. ^108^. **f** Reprinted with permission from ref. ^109^. **g** Reprinted with permission from ref. ^110^. **h** Reprinted with permission from ref. ^111^. **i** Reprinted with permission from ref. ^112^. **j** Reprinted with permission from ref. ^113^. **k** Reprinted with permission from ref. ^114^

Distinguished from the typical THz emission induced by optical rectification and photon-drag effect, the THz high-order harmonic generation (HHG) reflects the nonlinear process beyond second-order in materials including graphene, TMDs, Dirac semimetals, and topological insulators. For instance, an incident quasi-monochromatic, linearly polarized pump wave of 0.3 THz will cause harmonic emissions up to the seventh-order from the monolayer graphene at room temperature and under ambient conditions (Fig. 6a)^106^. In principle, the multiplication of photon energy is driven by the nonlinear interaction between light and matter. Free background Dirac electrons in graphene feature nonlinear intraband THz conductivity originated from the ability of internal thermalization and plays a role as an energy reservoir that promotes the light-to-matter energy transfer process. The nonlinear conductivity consequently results in modulated THz absorption and current in response to the pumping wave, leading to THz harmonic re-emission at multiplicated frequencies. On account of the centrosymmetric nature of graphene, only odd THz harmonics are generated. Moreover, parameter-free calculations using a thermodynamic model verify the high-harmonic generation efficiency and illustrate the potential for up to 13th THz harmonics (Fig. 6b)^106^. Particularly, the key to high-efficiency HHG emission is introducing and regulating the background carriers. By applying a gated voltage to graphene, the Dirac point and the background carrier density can be adjusted to the appropriate value, thus achieving the best HHG emission efficiency by striking a balance between enhancing the power absorption of the THz drive field and avoiding the nonlinear thermodynamic decline (Fig. 6c)^107^. Through this method, an increase of about two orders of magnitude in transmission efficiency has been achieved (Fig. 6d)^107^.

In addition to graphene, THz high-order harmonic generation is also observed from other Dirac semimetals such as Cd_3_As_2_. With the nonlinear kinetics of electron distribution and linear energy-momentum dispersion, electrons in the Dirac cone are accelerated and scattered by THz electric fields, accumulating energy into the electron subsystem, leading to a stretched and shifted distribution along the field, resulting in efficient HHG (Fig. 6e)^108^. Specifically, the HHG from Dirac semimetals exhibits non-perturbative fluence dependence and is predicted to be highly sensitive to the scattering rate. Nevertheless, the HHG characteristics of Cd_3_As_2_ are reported to be related to the supercritical or subcritical regime of nonlinear optics, corresponding to whether all electrons can be driven beyond the Dirac point^109^. In the supercritical regime, up to the 31st order HHG can be generated using a modestly powered driving laser (Fig. 6f). In contrast, the subcritical regime leads to zero intraband emission of 5th and higher order HHG. More recently, the regulation of third-harmonic yield and polarization state in Cd_3_As_2_ through driving-pulse ellipticity has been achieved (Fig. 6g)^110^. The increased ellipticity results in enhanced perpendicular component and decreased parallel component of the emitted electric field through nonlinear intraband kinetic processes, indicating the THz optical signal processing potential of Dirac semimetals.

Besides, as a type of Dirac material, topological insulators (TI) such as Bi_2_Te_3_ exhibit interesting THz HHG characteristics. Innovatively, tunable non-integer HHG is demonstrated in Bi_2_Te_3_ film^111^. With the ballistic acceleration of Dirac currents in the topologically protected surface state, HHG dominated by the bulk or the surface state can be selected by tuning the driving frequency (Fig. 6h). When the carrier-envelope phase of the driving field changes, the special dynamics enables a continuous shift of HHG to arbitrary non-integer multiples of the driving frequency. Furthermore, topological insulators demonstrate intriguing feature of enhanced THz HHG efficiency. In topological insulator Bi_2_Se_3_, third-order THz harmonics at milliwatt level with a field conversion efficiency of ~8% is achieved, which is nearly two orders of magnitude better than monolayer graphene at a high incident power (Fig. 6i)^112^. The enhanced emission is attributed to the ultrafast dissipation of electronic heat through the surface states of the bulk and thus avoiding heat accumulation in both electronic and phononic systems. Moreover, using a metal grating combined with TI can increase the harmonic power from low to intermediate incident powers by surface-selective facilitating. Apart from TI, THz HHG can also be generated in 2D semiconductors such as TMDs. In MoS_2_, up to the 13th-order harmonic can be observed when excited by incident light of 0.3 eV (72.55 THz)^113^. Due to the non-centrosymmetric nature of its lattice, even-order harmonics also appear (Fig. 6j). Compared to bulk MoS_2_ materials, the enhanced odd HHG efficiency is considered relative to the reduced dielectric screening and strong electron-hole Coulomb interaction in the monolayer.

Clarification of the nonperturbative electron dynamics under low-frequency and high-intensity fields in these Dirac systems is of great significance. Recently, an analytical approach based on the (Jeffreys-)Wentzel-Kramers-Brillouin (WKB) approximation has been proposed to analyze HHG in the THz regime for realistic massive Dirac materials, which provides HHG waveforms that match well with the numerical results (Fig. 6k)^114^. THz HHG in the material system with Dirac electronic properties provides additional emission frequencies and offers valuable insight on the intraband dynamics of Dirac electrons. However, the frequencies and relative intensities of the harmonics are usually limited to multiples of the excitation frequency and magnitudes of the incident power several orders below, which requires further exploration to expand its availability for THz source applications.

## THz emission arising from spin-to-charge conversion

Different from the mechanism that requires the symmetry-breaking either by the non-centrosymmetric lattice structure or by the asymmetrical geometry of the photoexcitation, a novel mechanism known as spin-to-charge conversion has emerged as an alternative pathway for THz emission in heterostructures comprising a solitary ferromagnetic (FM) layer alongside a non-magnetic (NM) layer. In such heterostructures, the spin-orbit coupling at the FM/NM interface plays a crucial role in the spin-to-charge conversion process. In brief, while an ultrafast laser pulse is incident on the heterostructure, it generates a non-equilibrium distribution of spin-polarized charge carriers in the FM layer, wherein the spins of these carriers are coupled to their momentum due to the spin-orbit coupling at the FM/NM interface. This coupling leads to the generation of a pure spin current in the FM layer, which propagates across the FM layer until it reaches the FM/NM interface. At this interface, the spin current can undergo a conversion into a charge current, leading to the emission of THz radiation. In essence, this spin-to-charge conversion process occurs due to the transfer of angular momentum between the spin and the charge degrees of freedom at the interface.

**Fig. 7:**
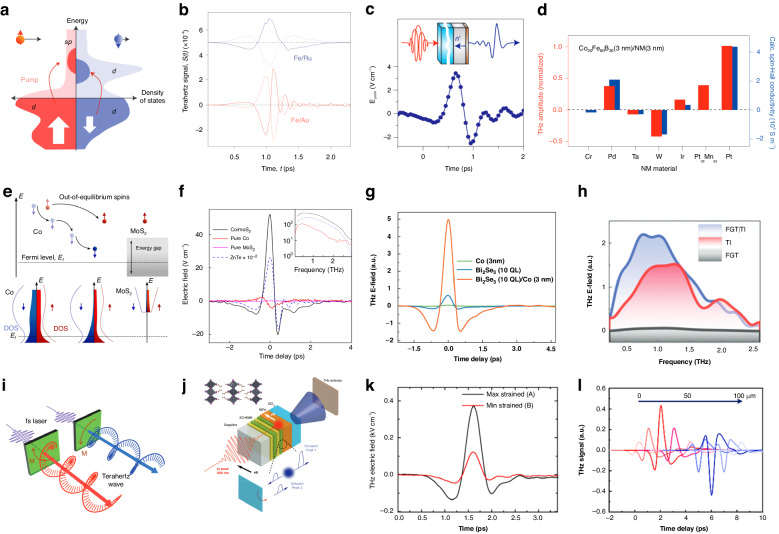
THz emission from spin-to-charge conversion. **a** Non-equilibrium electron distribution in the iron layer, excited by a femtosecond laser pulse. **b** THz emission from Fe/Au and Fe/Ru heterostructures, of which the signal inverts with reversal of the sample magnetization. **c** THz emission from Co/Pt heterostructure, with the pump is incident from the side of the substrate. **d** Impact of different NM layer materials on the THz output amplitude of the FM/NM spintronic emitters. **e** Schematic and the electron population change in the spin injection process from Co to monolayer MoS_2_. **f** THz emission from Co/MoS_2_ heterostructure, compared with pure Co, monolayer MoS_2_ and ZnTe crystal. **g** THz emission from Bi_2_Se_3_/Co heterostructure, pure Co and Bi_2_Se_3_. **h** THz emission from FGT(4 nm)|TI(8 nm), FGT(4 nm), and TI(8 nm) in frequency domain. **i** Manipulation of the THz chirality by controlling the magnetic field (Schematic illustration). **j** Asymmetric spintronic-THz emission from Pt/NiFe and 2D-HMH/NiFe heterostructures (Schematic illustration). **k** Electric-field-tunable THz spintronic emission under maximum and minimum strained states. **l** THz emission waves with manipulated amplitudes and polarities. **a**, **b** Reprinted with permission from ref. ^102^. **c** Reprinted with permission from ref. ^115^. **d** Reprinted with permission from ref. ^116^. **e**, **f** Reprinted with permission from ref. ^117^. **g** Reprinted with permission from ref. ^118^. **h** Reprinted with permission from ref. ^119^. **i** Reprinted with permission from ref. ^120^. **j** Reprinted with permission from ref. ^121^. **k** Reprinted with permission from ref. ^122^. **l** Reprinted with permission from ref. ^123^

Such spintronic THz emission is observed from metallic FM/NM heterostructures in 2013. When Fe/Au or Fe/Ru heterostructures are incident with a femtosecond laser pulse at 1.55 eV, the absorption in the Fe layer made the majority-spin electrons in iron with mainly sp-like character excited, the non-equilibrium electron distribution then leads to immediate spin current (Fig. 7a), resulting in THz emission through the inverse spin Hall effect^102^. The THz emission polarizes perpendicularly with respect to the magnetization, covering the bandwidth from 0.3 ~ 20 THz, which is tunable by adjusting the non-magnetic layer material (Fig. 7b). Especially, excited by circularly polarized optical pulses, similar FM/NM heterostructures composed of NM material with strong spin-orbit interaction exhibit helicity-dependent THz emission characteristics (Fig. 7c). For example, Co/Pt heterostructure can generate laser-induced THz radiation parallel to the magnetization M (E_y_)^115^, which is distinguished from the pump polarization independent spintronic emission induced by the inverse spin Hall effect (E_x_). The maximum amplitude of E_y_ is typically one to two orders of magnitude smaller than E_x_, and hardly change with the thickness of the metal layer, indicating a spin-orbit interaction at the contact interface. Notably, the sign of the emitted THz radiation will change if either the helicity of the circularly polarized light reverses or the heterostructure is rotated around the magnetization over 180° (Fig. 7c), revealing that the heterostructures act as an electric-dipole origin with space-inversion symmetry breaking.

The output of the STE can be optimized by designing the layer materials and multilayer film structures. For the FM/NM metal heterostructure, material of the NM layer significantly alters the THz amplitude, as shown in Fig. 7d. Among the various NM materials studied, the output intensity is not strictly correlated with the material’s spin Hall conductivity^116^. Interestingly, W with a half-filled d-electron shell results in a relatively large amplitude in an opposite direction. Exploiting this, placing two NM thin films with opposite spin Hall angles (W and Pt for instance) on either side of the FM layer enables the forward and backward spin currents to eventually convert into in-phase radiation, significantly improving the efficiency of pump energy utilization. As for the FM layer, the influence on the output amplitude is relatively small when different materials are used, among which the CoFeB film has been identified as a more optimal solution. Ultimately, the optimized W/CoFeB/Pt three-layer spin emitter can generate pulses covering the 1–30 THz range without a gap, with amplitudes surpassing standard emitters such as ZnTe, GaP, and biased photoconductive switches.

Apart from metal heterostructures, the challenge of giant spin injection into semiconductors is resolved in a Co/2D semiconductor MoS_2_ heterostructure, leading to THz emission induced by spin-to-charge conversion^117^. As the out-of-equilibrium carrier distribution in the ferromagnetic layer diffuses and relaxes in energy states, almost only the majority-spin electrons remain at high energies, which are then filtered by the band gap of MoS_2_ and result in a large spin injection effect (Fig. 7e). Throughout this process, the strong spin-orbital interaction of MoS_2_ enables efficient THz emission induced by spin-to-charge conversion (Fig. 7f). Particularly, full parameter-space characterization shows that the THz amplitude has a spin-current-based origin, and is independent of the orientation-related nonlinear optical response. The peak THz intensity is regulated by pump photon energy, indicating a non-thermalized process in mechanism. Additionally, spin-to-charge conversion is realized in Bi_2_Se_3_/Co heterostructure, which is attributed to the important role of spin-momentum locked surface states in the topological insulator^118^. THz emission of the heterostructure is very apparent compared with the emission of either Bi_2_Se_3_ or Co film (Fig. 7g), indicating a high efficiency of spin-injection and spin-to-charge conversion. The special properties of 2D materials can also be exploited in the ferromagnetic layer. 2D vdW ferromagnetic Fe_3_GeTe_2_ (FGT) has been used to construct FM/TI heterostructures^119^. FGT integrated with Bi_2_Te_3_ exhibits spin-to-charge conversion-induced THz emission through the inverse Edelstein effect mechanism (Fig. 7h), regulated by the direction of the magnetic field, the pumping incidence, and the thickness of the two films. It is noteworthy that the interfacial exchange coupling in the FGT/TI heterostructure is observed to stabilize the ferromagnetic order of 2D FGT, providing insights into low-dimensional characteristics of layered FM materials.

For device applications in THz communication, modulation of amplitude, phase, polarization, etc. is significant for optimized transmission efficiency. Inspired by the linear polarization of spintronic THz emission from FM/NM structure, a nonuniform external magnetic field is designed to realize THz wave generation and tuning of the chirality (Fig. 7i)^120^. The azimuthal angle can be manipulated by rotating the sample and the magnets. Additionally, the ellipticity can be tuned by changing the phase difference or the emitting areas, and also depends on the frequency. However, strict tuning of the phase difference for circularly polarized emission is still challenging, requiring further exploration in adjusting the material thickness and conductivity of the FM/NM nanofilms.

Coherent control of THz radiation is demonstrated in 2D hybrid metal halides (2D-HMH)/NiFe heterostructures^121^. As an emerging type of synthetic semiconductor, 2D-MHM features spin-orbit coupling due to the periodically layered inorganic PbI_6_ framework structure, revealing the intriguing potential for STE. Through an inverse Rashba-Edelstein effect mechanism extended with additional in-plane momentum shift of the Rashba bands, the phase and intensity are manipulated by tuning the applied magnetic field and linear polarization of the femtosecond laser pump pulses (Fig. 7j). This dependence is not observed in Pt/NiFe or 3D-HMH/NiFe devices, indicating a low-dimensionality related origin of the 2D-HMH layer under below-gap femtosecond laser excitation. Moreover, by integrating Pt/CoFe heterostructure with a single crystal piezoelectric Pb(Mg_1/3_Nb_2/3_)O_3_–PbTiO_3_ (PMN–PT) layer, an artificial magnetoelectric coupling is endowed through strain transfer across the ferroelectric (FE)/FM interface, thus realizing an effective electric-field modulation of 270% of the THz amplitude (Fig. 7k)^122^.

In addition to exploiting material properties, surface-patterned structural design is used for spintronic THz modulation and coded emission. Combining interspaced Co/Pt and Co/W heterostructures with an optical mask enables selective excitation of micro emitters with opposite spin Hall angles, resulting in coded THz phase components (Fig. 7l)^123^. Such spatial design provides an additional operational dimension, demonstrating the direction of more integrable and multifunctional devices.

Studying THz emission from heterostructures with FM and NM layers allows researchers to explore spin dynamics, spin transport, and spin-related phenomena. Meanwhile, the inclusion of extra spin degrees of freedom and the utilization of diverse FM/NM material systems broaden the possibilities for generating and modulating THz emissions, offering potential applications in spintronic THz devices.

## Extended THz sources and their applications

**Fig. 8:**
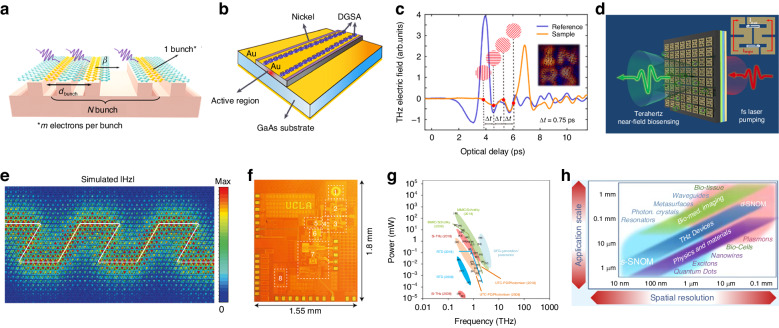
Extended THz sources and their applications. **a** Schematic diagram of the emitter based on Smith–Purcell Radiation. **b** Three-dimensional schematic of the laser structure with the DGSA integrated onto the gold top layer contact of the QCL waveguide. **c** THz waveforms acquired by single-shot ultrafast photography system, four asynchronously arriving sub-pulses are used to obtain time-resolved images (inset). **d** Near-field THz sensing of Hela cells and Pseudomonas based on monolithic integrated metamaterials with STE. **e** Simulated z-oriented magnetic field distribution (color scale) in the on-chip valley Hall photonic crystal at 0.335 THz. The white line denotes the position of the domain wall. **f** Die photo of THz frequency synthesizer systems with fully integrated THz phase-locked loops in CMOS. **g** Progress of THz sources, exhibiting the development of specific THz technology in producing continuous-wave THz signals at room temperature. **h** Overview diagram of THz near-field imaging and spectroscopy applications. **a** Reprinted with permission from ref. ^124^. **b** Reprinted with permission from ref. ^125^. **c** Adapted with permission from ref. ^126^. **d** Reprinted with permission from ref. ^127^. **e** Reprinted with permission from ref. ^128^. **f** Reprinted with permission from ref. ^129^. **g** Reprinted with permission from ref. ^130^. **h** Reprinted with permission from ref. ^15^

Beyond THz generation induced by femtosecond optical excitation, the utilization of various mechanisms and structures is also being thoroughly explored. For instance, the Smith-Purcell effect arises when charge carriers move on periodically patterned surfaces, leading to THz emission. However, traditional SP emitters are constrained by electron beam acceleration and alignment. Recently, an SP THz emitter utilizing charge carriers in 2D materials has been designed^124^. Numerical simulations indicate that coherent THz radiation can be expected by integrating graphene and silicon grating and exciting the hot electrons within (Fig. 8a). The performance of this emitter holds the potential for further modulation through experimental transformations of expanded 2D materials and the design of specific metasurface structures. It represents a promising THz source with potential for high efficiency, near-field excitation, and on-chip integration adaptability.

In comparison to THz time-domain systems, QCLs exhibit more availability as miniaturized direct laser sources, which provides a compact solution with chip-scale dimensions, high power emission, high spectral purity, and wide bandwidth. However, engineering intracavity semiconductor multilayers with the necessary gain and absorption properties at THz frequencies presents significant challenges due to the extremely low photon energies involved. Riccardi et al.^125^ demonstrated the generation of 4 ps pulses in a semiconductor heterostructure THz laser using passive mode-locking. A distributed graphene saturable absorber (DGSA) was produced by transferring a seven-layer CVD-grown multilayer graphene (MLG) film along the entire cavity of double-metal QCL (Fig. 8b). Commercial femtosecond systems in the visible/infrared spectrum rely on passive pulse generation due to their ability to generate the shortest and most powerful pulses. Consequently, this research offers promising prospects for spectroscopy applications.

Due to its unique frequency characteristics, THz light sources exhibit distinct advantages in fields such as ultrafast imaging, biosensing, and high-bandwidth communication. Leveraging the penetrative nature of THz radiation, a single-shot ultrafast imaging system has been realized^126^. The initial probing beam is divided into multiple sub-pulses differentiated in the frequency domain with varying delay times, thus achieving time-resolved image acquisition (Fig. 8c). Through the composite detection, decoding, and reconstruction in both time and spatial frequency domains, this system possesses the capability to capture transient events within non-transparent media, achieving a time resolution at the sub-picosecond scale. This method extends the limitations of ultrafast photography based on traditional wavelength light source, providing a powerful tool for exploring fundamental dynamic events in various domains including physics, chemistry, biology, materials, etc.

Moreover, implementing label-free biosensors in the THz spectra has enabled many potential applications, such as bio-material detection in point-of-care (PoC) diagnostics. Bai et al.^127^ conducted a study in which they exhibited a monolithic THz emission biosensor integrating asymmetric double-split ring resonator metamaterials with a ferromagnetic heterojunction STE (Fig. 8d). This THz emission biosensor has enormous potential for high-resolution near-field biosensing applications, especially for detecting trace biological samples.

The advancement of THz-frequency waveguides is necessary for developing integrated, cost-effective, and efficient solutions for high-speed devices. Yang et al.^128^ demonstrated the robust transport of THz topological valleys through multiple sharp bends on an all-silicon chip to surmount these obstacles using full-wave simulations and visualized the intensity distribution of the magnetic field near the twisted domain wall (Fig. 8e). The THz topological photonic circuit is immune to sharp bends, and is able to achieve error-free transmission of uncompressed 4 K high-definition video, demonstrating the immense potential of THz topological photonics in applications such as topological splitters, robust delay lines, compact interferometers, and directional antennas.

To address integrated application scenarios, THz emission or detection can be described in the logic way of electronic and photonic way, which serves as the basis for THz technology. Figure 8f^129^ demonstrates that TSMC has accomplished a remarkable feat by effectively fabricating a circuit using standard 65 nm CMOS technology with a die size of 1.55 × 1.80 mm^2^. Several crucial techniques, including incorporating band-selection inductor switches, simultaneous bulk voltage tuning across oscillators and dividers, a mutual injection locking mechanism, and a dual port injection divider, have been implemented to achieve a locking range of 21 GHz.

Significant advances have been made in developing THz signal generation technologies over the recent years (Fig. 8g). In the field of electronics, III-V-based semiconductor technologies have demonstrated the capacity to generate 100 $\mu$W to mW range of power at room temperature^130^, representing a two- to five-fold increase, especially at frequencies above 1 THz. Similarly, photonic approaches to THz generation have made significant strides. Photomixer-based methods have achieved continuous-wave power levels in the tens of microwatt range. In contrast, difference-frequency or parametric generation utilizing nonlinear materials has achieved power levels in the 1–2 THz range, reaching the milliwatt range.

In the quest for extending the scale of THz phenomena investigation beyond the diffraction limit, significant attention is directed towards the synergistic integration of THz radiation with scanning near-field optical microscopy (SNOM). This technique is commonly categorized as d-SNOM assisted by subwavelength probe, and s-SNOM enhanced by a sharp tip. At different spatial resolutions and scales, the application space of near-field THz imaging includes bio-med imaging, THz devices, physics and materials, etc. (Fig. 8h). Stem from the long wavelength nature of THz radiation, the confinement of THz radiation to sub-wavelength scales and accomplishing nonlinear near-field spectroscopy in the THz range are the main challenges unresolved^15^. Breakthroughs in these limitations will propel advancements in THz research, particularly in the areas of cryogenic temperatures, ultrafast timescales, and nanoscale lengths.

## Summary and future perspectives

To encapsulate, THz emission spectroscopy has proven to be a powerful and versatile tool for investigating the electronic and optical properties of 2D (or quasi-2D) materials and their interfaces. This review has explained the diverse nonlinear processes underlying THz emission, including optical rectification, photon-drag effect, interfacial processes, high-order THz harmonics generation, and spin-to-charge conversion, showcasing the rich diversity of THz emission spectroscopy in unraveling the complex nature of these materials. These findings contribute to the understanding of novel 2D (or quasi-2D) materials and pave the way for advancements in fields ranging from materials science to photonics, optoelectronics, and quantum technologies. From the perspective of technological application, these 2D (or quasi-2D) materials have shown huge potential to be next-generation laser-driven table-top THz emitters due to their atomically thin structure and easy integration into silicon wafer to make compact devices. Although these different 2D material and their structures can emit THz radiation under fs laser illumination, the real application using them for THz spectroscopy demands high efficiency and broad bandwidth to extract the THz response over a large range of spectrum. Of all the materials and structures mentioned earlier, STE stand out with significant advantages in terms of both high efficiency and a wide spectral range. Several strategies can be adopted to design the STE. First of all, the bilayer structure FM/NM structure can be replaced by a trilayer NM_1_/FM/NM_2_ structure^116^, where the NM_1_ and NM_2_ have opposite spin-hall angles. Secondly, multilayer structure (NM_1_/FM/NM_2_)_n_ can be made to enhance the THz emission. Thirdly, most of the femtosecond laser pulse are wasted due to the transmission and reflection at interfaces, whereas photonic crystal can be used to confine the laser electric field and strengthen the interaction between the laser and the metallic layer^131^. For example, constructing a simple Fabry-Pérot cavity is proved effective. The STE is sandwiched between a MgO layer and dielectric mirror [SiO_2_(165 nm)|TiO_2_(94 nm)]_5_, which confines the optical filed inside the cavity and minimizes pump transmission into Si and reflection into air^131^. Fourthly, special design such as THz anti-reflection film can be used to further enhance of the THz transmission. These strategies can be utilized either individually or in combination to attain maximum THz emission in the far field. Building upon this, explorations including the introduction of diverse 2D materials with strong spin-orbit interactions^132^, along with the complex structural control of the emitted THz waves, represents a frontier exploration in this field^133^.

However, there are still remaining challenges that need to be addressed to fully unlock the potential of THz spectroscopy of emerging 2D materials and heterostructures. This summary investigates the current problems and future development trends of THz spectroscopy, with a focus on achieving Spatial resolution, broadband spectral coverage, high signal-to-noise ratio, high efficiency and integrability. First of all, one primary challenge in THz spectroscopy is spatial resolution, as many emerging materials, particularly 2D materials, often exhibit nanoscale features that are significantly smaller than the diffraction-limited spot size of the far-field THz probe (~300 µm). As a result, far-field THz spectroscopy is limited to spatially-averaged measurements, providing information across inhomogeneous regions or ensembles. Despite the availability of near-field techniques discussed before, it remains difficult to achieve nanometer-scale resolution and probe individual nanoparticles or the internal structure of materials. Secondly, it is important to achieve efficient broadband coverage for THz generation and detection, as traditional THz sources and detectors often have limited frequency ranges, the necessity for wide-ranging measurements spanning from 0.1 to 30 THz is evident, as it covers the extensive frequency range encompassing spectral signatures in emerging materials. Additionally, the spectral characteristics may vary with experimental conditions and material geometry as discussed before. The THz conductivity spectra of emerging materials are frequently broadened due to high charge carrier scattering rates, further emphasizing the requirement for broadband sensitivity. To address this challenge, researchers are exploring novel sources and detectors such as QCLs, frequency combs, and metamaterial-based devices, which offer broader spectral coverage and higher resolution^134–139^. To expand the bandwidth and achieve sufficient signal strength up to at least 30 THz, further advancements are required, particularly in the development of spintronic^140^ and air-based^141^ THz generation and detection techniques. Broadband THz spectroscopy can enable researchers to explore a wider range of materials and physical phenomena with higher precision in the future. Another crucial point for THz technology is to increase the signal-to-noise ratio of THz emission. Enhancements in measurement sensitivity are necessary due to the weak absorption of THz radiation by many new materials. Additionally, THz measurements often require trade-offs between bandwidth, signal-to-noise ratio, acquisition speed, and spatial resolution. While increasing the intensity of the THz pulse can improve the signal-to-noise ratio, it can also introduce non-linear and non-equilibrium effects, making the probing process less minimally invasive. Thus, there is a need for novel techniques to improve the signal-to-noise ratio without compromising other measurement parameters.

On the other hand, high efficiency is an important factor in THz spectroscopy, as it directly influences the signal-to-noise ratio and overall performance of the system. Enhancing the efficiency of THz sources and detectors is crucial to improve the sensitivity and reliability of measurements. To this end, researchers are exploring various approaches, including optimizing device structures, enhancing conversion efficiencies, and integrating advanced materials and technologies, to achieve higher efficiency in THz spectroscopy systems^142–148^. It is worth noting that a recent spintronic heterostructure has demonstrated both a wide THz bandwidth (covering 0.3–30 THz) and high peak electric fields surpassing 1.5 MV/cm. This development holds significant potential in replacing the conventional approach of using LiNbO_3_ with tilted pulse-front configuration for generating strong THz fields, offering a more efficient and practical solution^131^. Additionally, a triple-layered chiral structure metasurface was proposed for high-efficiency broadband cross-polarization conversion in THz region, which can reach complete transmission phase coverage (0–2π) at a broadband frequency (0.4–1.0 THz), with 85.7% fractional bandwidth^142^, revealing the potential of integrating metasurfaces and heterogeneous structures for enhanced THz emission efficiency. Similarly, the various metasurfaces and interfaces in heterostructures based on 2D materials probably have great prospects for improving efficiency in the future. Besides, integrability is another key aspect for the practical implementation of THz technology, including trade-offs between various substrate technologies, limitations of device-level metrics, sensitivity to parasitics and packaging issues^130^. Seamless integration of THz sources, detectors, and other components into compact and robust devices would enable portable and field-deployable THz systems for communicating and imaging applications. Integration approaches such as photonic integration, microelectromechanical systems (MEMS), and on-chip technologies are being investigated to achieve compact and integrated THz spectroscopy systems^149–152^.

In conclusion, the future of THz spectroscopy technology is centered on overcoming key challenges, including achieving broad coverage, high-power operation, high efficiency, and integrability. To address these challenges, there is a need for advancements in THz source and detector technologies, as well as the exploration of innovative materials, heterostructures, and integration approaches, which is driving the development of more versatile THz spectroscopy systems. Furthermore, there is great potential for future 2D materials in THz emission, which encompasses leveraging 2D materials for devices like QCLs to tailor sub-levels for the THz range, along with exploring innovative structures such as 2D materials-photonic crystal heterostructures and Moiré superlattices. These advancements in THz spectroscopy not only expand our understanding of materials and physical phenomena but also open up a wide range of applications in fields such as materials science, biomedical imaging, physics, and communication.

## References

[1] Banks PA, Kleist EM, Ruggiero MT (2023). Investigating the function and design of molecular materials through terahertz vibrational spectroscopy. Nat. Rev. Chem..

[2] Köhler R (2002). Terahertz semiconductor-heterostructure laser. Nature.

[3] He XY (2022). 3D Dirac semimetals supported tunable terahertz BIC metamaterials. Nanophotonics.

[4] Cheng Y (2023). 3D Dirac semimetal supported thermal tunable terahertz hybrid plasmonic waveguides. Opt. Express.

[5] Wang GQ, Cao WH, He XY (2023). 3D Dirac semimetal elliptical fiber supported THz tunable hybrid plasmonic waveguides. IEEE J. Sel. Top. Quantum Electron..

[6] Fisher A (2022). Single-pass high-efficiency terahertz free-electron laser. Nat. Photonics.

[7] Auston DH (1984). Cherenkov radiation from femtosecond optical pulses in electro-optic media. Phys. Rev. Lett..

[8] Fattinger C, Grischkowsky D (1988). Point source terahertz optics. Appl. Phys. Lett..

[9] Hu BB, Nuss MC (1995). Imaging with terahertz waves. Opt. Lett..

[10] O’Hara J, Grischkowsky D (2001). Quasi-optic terahertz imaging. Opt. Lett..

[11] Tonouchi M (2007). Cutting-edge terahertz technology. Nat. Photonics.

[12] Zeitler JA, Gladden LF (2009). In-vitro tomography and non-destructive imaging at depth of pharmaceutical solid dosage forms. Eur. J. Pharmaceutics Biopharmaceutics.

[13] Jepsen PU, Cooke DG, Koch M (2011). Terahertz spectroscopy and imaging—modern techniques and applications. Laser Photonics Rev..

[14] Amini T (2021). A review of feasible applications of THz waves in medical diagnostics and treatments. J. Lasers Med. Sci..

[15] Leitenstorfer A (2023). The 2023 terahertz science and technology roadmap. J. Phys. D: Appl. Phys..

[16] Khan K (2020). Recent developments in emerging two-dimensional materials and their applications. J. Mater. Chem. C.

[17] Chang C (2021). Recent progress on two-dimensional materials. Acta Phys. Chim. Sin..

[18] Zhang ZW (2017). Robust epitaxial growth of two-dimensional heterostructures, multiheterostructures, and superlattices. Science.

[19] Prabhu P, Jose V, Lee JM (2020). Design strategies for development of TMD-based heterostructures in electrochemical energy systems. Matter.

[20] Jin J (2021). Hierarchical MXene/transition metal chalcogenide heterostructures for electrochemical energy storage and conversion. Nanoscale.

[21] Kim G, Song S, Jariwala D (2023). Spatially controlled two-dimensional quantum heterostructures. Mater. Res. Lett..

[22] Sun JW (2021). Strong plasmon-exciton coupling in transition metal dichalcogenides and plasmonic nanostructures. Nanoscale.

[23] Duan XD (2014). Lateral epitaxial growth of two-dimensional layered semiconductor heterojunctions. Nat. Nanotechnol..

[24] Guo YZ, Robertson J (2016). Band engineering in transition metal dichalcogenides: stacked versus lateral heterostructures. Appl. Phys. Lett..

[25] Ko KY (2018). High-performance gas sensor using a large-area WS_2*x*_Se_2-2*x*_ alloy for low-power operation wearable applications. ACS Appl. Mater. Interfaces.

[26] Lee CH (2022). Design of p-WSe_2_/n-Ge heterojunctions for high-speed broadband photodetectors. Adv. Funct. Mater..

[27] Chen YF (2022). Momentum-matching and band-alignment van der Waals heterostructures for high-efficiency infrared photodetection. Sci. Adv..

[28] Li D (2022). Electronic gap characterization at mesoscopic scale via scanning probe microscopy under ambient conditions. Nat. Commun..

[29] Nugera FA (2022). Bandgap engineering in 2D lateral heterostructures of transition metal dichalcogenides via controlled alloying. Small.

[30] Du W (2019). Ultrafast modulation of Exciton–Plasmon coupling in a monolayer WS_2_–Ag nanodisk hybrid system. ACS Photonics.

[31] Duan XD (2015). Two-dimensional transition metal dichalcogenides as atomically thin semiconductors: opportunities and challenges. Chem. Soc. Rev..

[32] Wang, C., Huang, Y. & Duan, X. F. Enhanced electrical characteristics of black phosphorus by polyaniline and protonic acid surface doping. In: *Proc. IEEE 17th International Conference on Nanotechnology* 453–455 (IEEE, 2017) 10.1109/NANO.2017.8117384.

[33] Zhang SM (2022). Lateral layered semiconductor multijunctions for novel electronic devices. Chem. Soc. Rev..

[34] Tan, W. C. et al. in *2D Semiconductor Materials and Devices* (eds Chi, D., Goh, K. E. J. & Wee, A. T. S.) 251–312 (Elsevier, 2020).

[35] Wu H (2019). A field-effect approach to directly profiling the localized states in monolayer MoS_2_. Sci. Bull..

[36] Huang GY (2023). All-optical reconfigurable excitonic charge states in monolayer MoS_2_. Nano Lett..

[37] Zheng D (2017). Manipulating coherent plasmon-exciton interaction in a single silver nanorod on monolayer WSe_2_. Nano Lett..

[38] Wang C (2018). Monolayer atomic crystal molecular superlattices. Nature.

[39] Kong W (2019). Path towards graphene commercialization from lab to market. Nat. Nanotechnol..

[40] Geim AK, Grigorieva IV (2013). Van der Waals heterostructures. Nature.

[41] Liu Y, Huang Y, Duan XF (2019). Van der Waals integration before and beyond two-dimensional materials. Nature.

[42] Kim KS (2023). Non-epitaxial single-crystal 2D material growth by geometric confinement. Nature.

[43] Zhou ZJ (2023). Stack growth of wafer-scale van der Waals superconductor heterostructures. Nature.

[44] Liu, C. et al. Controllable van der Waals gaps by water adsorption. *Natu. Nanotechnol.* (in the press). 10.1038/s41565-023-01579-w 38177277

[45] Jayachandran D (2024). Three-dimensional integration of two-dimensional field-effect transistors. Nature.

[46] Wu F (2022). Vertical MoS_2_ transistors with sub-1-nm gate lengths. Nature.

[47] Zhu KC (2023). Hybrid 2D-CMOS microchips for memristive applications. Nature.

[48] O’Brien KP (2023). Process integration and future outlook of 2D transistors. Nat. Commun..

[49] Shen Y (2022). The trend of 2D transistors toward integrated circuits: scaling down and new mechanisms. Adv. Mater..

[50] Joksas D (2022). Memristive, spintronic, and 2D-materials-based devices to improve and complement computing hardware. Adv. Intell. Syst..

[51] Zhu EB, Zhang ZW, Wang C (2023). Editorial: emerging chip materials and devices for post Moore’s era. Front. Mater..

[52] Goel N, Kumar M (2021). 2D materials for terahertz application. Nano Express.

[53] Huang YY (2018). Terahertz surface emission from layered MoS_2_ crystal: competition between surface optical rectification and surface photocurrent surge. J. Phys. Chem. C..

[54] Yao ZH (2018). Interface properties probed by active THz surface emission in graphene/SiO_2_/Si heterostructures. ACS Appl. Mater. Interfaces.

[55] Zheng W (2016). Optically pumped terahertz wave modulation in MoS_2_-Si heterostructure metasurface. AIP Adv..

[56] Ma EY (2019). Recording interfacial currents on the subnanometer length and femtosecond time scale by terahertz emission. Sci. Adv..

[57] Liu XJ (2020). Circular photogalvanic spectroscopy of Rashba splitting in 2D hybrid organic-inorganic perovskite multiple quantum wells. Nat. Commun..

[58] Kumar N (2015). Investigation of terahertz emission from BiVO_4_/Au thin film interface. J. Infrared Millim. Terahertz Waves.

[59] Leitenstorfer A (2000). Femtosecond high-field transport in compound semiconductors. Phys. Rev. B.

[60] Kushnir K (2017). Ultrafast zero-bias photocurrent in GeS nanosheets: promise for photovoltaics. ACS Energy Lett..

[61] Du WY (2020). Photodoping of graphene/silicon van der Waals heterostructure observed by terahertz emission spectroscopy. Appl. Phys. Lett..

[62] Bagsican FRG (2020). Terahertz excitonics in carbon nanotubes: exciton autoionization and multiplication. Nano Lett..

[63] Hangyo M, Nagashima T, Nashima S (2002). Spectroscopy by pulsed terahertz radiation. Meas. Sci. Technol..

[64] Dragoman D, Dragoman M (2004). Terahertz fields and applications. Prog. Quantum Electron..

[65] Bera A (2021). Review of recent progress on THz spectroscopy of quantum materials: superconductors, magnetic and topological materials. Eur. Phys. J. Spec. Top..

[66] Yamamoto K, Ishida H (1994). Optical theory applied to infrared spectroscopy. Vibrational Spectrosc..

[67] Beard MC, Schmuttenmaer CA (2001). Using the finite-difference time-domain pulse propagation method to simulate time-resolved THz experiments. J. Chem. Phys..

[68] Lee WJ (2015). Na-dependent ultrafast carrier dynamics of CdS/Cu(In, Ga)Se_2_ measured by optical pump-terahertz probe spectroscopy. J. Phys. Chem. C..

[69] Han P, Wang XK, Zhang Y (2020). Time-resolved terahertz spectroscopy studies on 2D Van der Waals materials. Adv. Optical Mater..

[70] Ohta K (2017). Probing charge carrier dynamics in porphyrin-based organic semiconductor thin films by time-resolved THz spectroscopy. J. Phys. Chem. B.

[71] Cocker TL (2013). An ultrafast terahertz scanning tunnelling microscope. Nat. Photonics.

[72] Cocker TL (2016). Tracking the ultrafast motion of a single molecule by femtosecond orbital imaging. Nature.

[73] Yoshida S (2019). Subcycle transient scanning tunneling spectroscopy with visualization of enhanced terahertz near field. ACS Photonics.

[74] Yoshida S (2021). Terahertz scanning tunneling microscopy for visualizing ultrafast electron motion in nanoscale potential variations. ACS Photonics.

[75] Pizzuto A (2021). Nonlocal time-resolved terahertz spectroscopy in the near field. ACS Photonics.

[76] Huber MA (2017). Femtosecond photo-switching of interface polaritons in black phosphorus heterostructures. Nat. Nanotechnol..

[77] Liewald C (2018). All-electronic terahertz nanoscopy. Optica.

[78] Pizzuto A, Ma PC, Mittleman DM (2023). Near-field terahertz nonlinear optics with blue light. Light Sci. Appl..

[79] Aghamiri NA (2019). Hyperspectral time-domain terahertz nano-imaging. Opt. Express.

[80] Huang YY (2017). Surface optical rectification from layered MoS_2_ Crystal by THz time-domain surface emission spectroscopy. ACS Appl. Mater. Interfaces.

[81] Splendiani A (2010). Emerging photoluminescence in monolayer MoS_2_. Nano Lett..

[82] Si KY (2018). Terahertz surface emission from layered semiconductor WSe_2_. Appl. Surf. Sci..

[83] Zhang LH (2019). Polarized THz emission from in-plane dipoles in monolayer tungsten disulfide by linear and circular optical rectification. Adv. Optical Mater..

[84] Fan ZY (2020). Terahertz surface emission from MoSe_2_ at the monolayer limit. ACS Appl. Mater. Interfaces.

[85] Yue XY (2023). Real-time observation of the buildup of polaron in α-FAPbI_3_. Nat. Commun..

[86] Maysonnave J (2014). Terahertz generation by dynamical photon drag effect in graphene excited by femtosecond optical pulses. Nano Lett..

[87] Shi LK (2021). Geometric photon-drag effect and nonlinear shift current in centrosymmetric crystals. Phys. Rev. Lett..

[88] Bahk YM (2014). Plasmon enhanced terahertz emission from single layer graphene. ACS Nano.

[89] Obraztsov PA (2014). Photon-drag-induced terahertz emission from graphene. Phys. Rev. B.

[90] Zhu L (2017). Enhanced polarization-sensitive terahertz emission from vertically grown graphene by a dynamical photon drag effect. Nanoscale.

[91] Zhu LP (2019). Circular-photon-drag-effect-induced elliptically polarized terahertz emission from vertically grown graphene. Phys. Rev. Appl..

[92] Hamh SY (2016). Helicity-dependent photocurrent in a Bi_2_Se_3_ thin film probed by terahertz emission spectroscopy. Phys. Rev. B.

[93] Song Q (2021). Intensity-tunable terahertz radiation from tin selenide. J. Lumin..

[94] Cheng L (2023). Giant photon momentum locked THz emission in a centrosymmetric Dirac semimetal. Sci. Adv..

[95] Zhang (2023). Generation and control of ultrafast circular photon drag current in multilayer PtSe_2_ revealed via terahertz emission. Adv. Optical Mater..

[96] Bala Murali Krishna M (2018). Terahertz photoconductivity and photocarrier dynamics in few-layer hBN/WS_2_ van der Waals heterostructure laminates. Semicond. Sci. Technol..

[97] Ryzhii V (2020). Far-infrared and terahertz emitting diodes based on graphene/black-P and graphene/MoS_2_ heterostructures. Opt. Express.

[98] Song FC (2022). Ultrafast drift current terahertz emission amplification in the monolayer WSe_2_/Si heterostructure. J. Phys. Chem. Lett..

[99] Yao ZH (2019). Interfacial THz generation from graphene/Si mixed-dimensional van der Waals heterostructure. Nanoscale.

[100] Yang J (2021). Identifying the intermediate free-carrier dynamics across the charge separation in monolayer MoS_2_/ReSe_2_ heterostructures. ACS Nano.

[101] Li SH, Li JS (2018). Terahertz modulator a using CsPbBr_3_ perovskite quantum dots heterostructure. Appl. Phys. B.

[102] Kampfrath T (2013). Terahertz spin current pulses controlled by magnetic heterostructures. Nat. Nanotechnol..

[103] Tong MY (2021). Enhanced terahertz radiation by efficient spin-to-charge conversion in Rashba-mediated Dirac surface states. Nano Lett..

[104] Meineke C (2022). Scalable high-repetition-rate sub-half-cycle terahertz pulses from spatially indirect interband transitions. Light Sci. Appl..

[105] Li X (2022). Highly efficient and ultrafast terahertz modulation in perovskite hybrid structure. ACS Appl. Electron. Mater..

[106] Hafez HA (2018). Extremely efficient terahertz high-harmonic generation in graphene by hot Dirac fermions. Nature.

[107] Kovalev S (2021). Electrical tunability of terahertz nonlinearity in graphene. Sci. Adv..

[108] Kovalev S (2020). Non-perturbative terahertz high-harmonic generation in the three-dimensional Dirac semimetal Cd_3_As_2_. Nat. Commun..

[109] Lim J (2020). Efficient generation of extreme terahertz harmonics in three-dimensional Dirac semimetals. Phys. Rev. Res..

[110] Germanskiy S (2022). Ellipticity control of terahertz high-harmonic generation in a Dirac semimetal. Phys. Rev. B.

[111] Schmid CP (2021). Tunable non-integer high-harmonic generation in a topological insulator. Nature.

[112] Tielrooij KJ (2022). Milliwatt terahertz harmonic generation from topological insulator metamaterials. Light Sci. Appl..

[113] Liu HZ (2017). High-harmonic generation from an atomically thin semiconductor. Nat. Phys..

[114] Taya H, Hongo M, Ikeda TN (2021). Analytical WKB theory for high-harmonic generation and its application to massive Dirac electrons. Phys. Rev. B.

[115] Huisman TJ (2016). Femtosecond control of electric currents in metallic ferromagnetic heterostructures. Nat. Nanotechnol..

[116] Seifert T (2016). Efficient metallic spintronic emitters of ultrabroadband terahertz radiation. Nat. Photonics.

[117] Cheng L (2019). Far out-of-equilibrium spin populations trigger giant spin injection into atomically thin MoS_2_. Nat. Phys..

[118] Wang XB (2018). Ultrafast spin-to-charge conversion at the surface of topological insulator thin films. Adv. Mater..

[119] Chen XH (2022). Generation and control of terahertz spin currents in topology-induced 2D ferromagnetic Fe_3_GeTe_2_|Bi_2_Te_3_ heterostructures. Adv. Mater..

[120] Kong DY (2019). Broadband spintronic terahertz emitter with magnetic-field manipulated polarizations. Adv. Opt. Mater..

[121] Cong KK (2021). Coherent control of asymmetric spintronic terahertz emission from two-dimensional hybrid metal halides. Nat. Commun..

[122] Agarwal P (2022). Electric-field control of nonlinear THz spintronic emitters. Nat. Commun..

[123] Tong MY (2022). Light-driven spintronic heterostructures for coded terahertz emission. ACS Nano.

[124] Lu SY (2023). Smith–Purcell radiation from highly mobile carriers in 2D quantum materials. Laser Photonics Rev..

[125] Riccardi E (2023). Short pulse generation from a graphene-coupled passively mode-locked terahertz laser. Nat. Photonics.

[126] Dong JL (2023). Single-shot ultrafast terahertz photography. Nat. Commun..

[127] Bai ZY (2020). Near-field terahertz sensing of HeLa cells and pseudomonas based on monolithic integrated metamaterials with a spintronic terahertz emitter. ACS Appl. Mater. Interfaces.

[128] Yang YH (2020). Terahertz topological photonics for on-chip communication. Nat. Photonics.

[129] Zhao Y (2016). A 0.56 THz phase-locked frequency synthesizer in 65 nm CMOS technology. IEEE J. Solid-State Circuits.

[130] Sengupta K, Nagatsuma T, Mittleman DM (2018). Terahertz integrated electronic and hybrid electronic–photonic systems. Nat. Electron..

[131] Rouzegar R (2023). Broadband spintronic terahertz source with peak electric fields exceeding 1.5 MV/cm. Phys. Rev. Appl..

[132] Abdukayumov, K. et al. Atomic-layer controlled THz spintronic emission from epitaxially grown two dimensional PtSe_2_/ferromagnet heterostructures. Preprint at https://arxiv.org/abs/2305.06895 (2023).

[133] Wang, S. J. et al. Flexible generation of structured terahertz fields via programmable exchange-biased spintronic emitters. Preprint at https://arxiv.org/abs/2311.11499 (2023).

[134] Fischer BM, Helm H, Jepsen PU (2007). Chemical recognition with broadband THz spectroscopy. Proc. IEEE.

[135] Blank V, Thomson MD, Roskos HG (2013). Spatio-spectral characteristics of ultra-broadband THz emission from two-colour photoexcited gas plasmas and their impact for nonlinear spectroscopy. N. J. Phys..

[136] Jeong JH (2013). High-power broadband organic THz generator. Sci. Rep..

[137] Zouaghi W (2013). Broadband terahertz spectroscopy: principles, fundamental research and potential for industrial applications. Eur. J. Phys..

[138] D’Angelo F (2014). Ultra-broadband THz time-domain spectroscopy of common polymers using THz air photonics. Opt. Express.

[139] Regensburger S (2015). Broadband THz detection from 0.1 to 22 THz with large area field-effect transistors. Opt. Express.

[140] Seifert TS (2022). Spintronic sources of ultrashort terahertz electromagnetic pulses. Appl. Phys. Lett..

[141] Tarekegne AT (2019). Terahertz time-domain spectroscopy of zone-folded acoustic phonons in 4H and 6H silicon carbide. Opt. Express.

[142] Fan JP, Cheng YZ (2020). Broadband high-efficiency cross-polarization conversion and multi-functional wavefront manipulation based on chiral structure metasurface for terahertz wave. J. Phys. D: Appl. Phys..

[143] Hooper IR (2019). High efficiency photomodulators for millimeter wave and THz radiation. Sci. Rep..

[144] Porterfield, D. W. High-efficiency terahertz frequency triplers. In: *Proc. 2007 IEEE/MTT-S International Microwave Symposium* 337–340 (IEEE, 2007).

[145] He T (2015). High-efficiency THz modulator based on phthalocyanine-compound organic films. Appl. Phys. Lett..

[146] Ouchi T (2014). Terahertz imaging system for medical applications and related high efficiency terahertz devices. J. Infrared Millim. Terahertz Waves.

[147] Bakhtiari F, Esmaeilzadeh M, Ghafary B (2017). Terahertz radiation with high power and high efficiency in a magnetized plasma. Phys. Plasmas.

[148] Ou HL (2020). Tunable terahertz metamaterial for high-efficiency switch application. Results Phys..

[149] Banerjee A (2019). Performance improvement of on-chip integrable terahertz microbolometer arrays using nanoscale meander titanium thermistor. J. Appl. Phys..

[150] Samanta D (2021). Tunable graphene nanopatch antenna design for on-chip integrated terahertz detector arrays with potential application in cancer imaging. Nanomedicine.

[151] Lu XY (2022). Integrated intelligent electromagnetic radiator design for future THz communication: a review. Chin. J. Electron..

[152] Sarkar P (2022). Review on the evolution of 6G and terahertz communication for highspeed information processing. Bull. Russian Acad. Sci.: Phys..

